# Supplementary material for: A generalizable and accessible approach to machine learning with global satellite imagery
Source: Nat Commun. 2021 Jul 20;12:4392. doi: 10.1038/s41467-021-24638-z (PMC8292408; doi:10.1038/s41467-021-24638-z)
Supplement: Supplementary file 1 — Supplementary Information [file 41467_2021_24638_MOESM1_ESM.pdf]

Supplementary Materials

A Generalizable and Accessible Approach to Machine Learning with Global Satellite Imagery

Esther Rolf, Jonathan Proctor, Tamma Carleton, Ian Bolliger, Vaishaal Shankar, Miyabi Ishihara, Benjamin Recht, Solomon Hsiang

The primary goal of our analysis is to develop, evaluate, and contextualize the performance of MOSAIKS. In the following three supplementary sections we first describe the data, then detail the methods behind the experiments conducted, and finally show how MOSAIKS compares to other approaches in the literature in greater depth. We also describe the intuition behind and the mechanics of MOSAIKS's algorithms in greater detail.

Contents

1 Supplementary Note 1: Data **2**

1.1 Labels 3

1.2 Imagery 11

2 Supplementary Note 2: Methods **12**

2.1 Grid definition and sampling strategy 12

2.2 Assigning labeled data to sampled imagery 13

2.3 Featurization of satellite imagery 15

2.4 Data separation practices and cross-validation 22

2.5 Training and testing the model 23

2.6 Primary model test set performance, robustness to functional form, and spatial distribution of errors 28

2.7 Altering the number of features and training set size 33

2.8 Testing generalizability across space and comparison to kernel-based interpolation. 34
2.9 Label super-resolution 37
2.10 Global model 45
2.11 Generalizing to other ACS variables 47

3 Supplementary Note 3: Comparisons to other models 48

3.1 Benchmarking performance 48
3.2 Comparing costs 60

1 Supplementary Note 1: Data

This Supplementary Note describes the datasets we use to construct our ground truth labels across all seven of our tasks: forest cover, elevation, population density, nighttime lights, income, road length, and housing price. In addition, we describe the imagery used in the analysis. In Note 2.2 we detail our method for linking the labeled data for each outcome to the imagery (Supplementary Figure 4).

In evaluating the ability of MOSAIKS to generalize, we are interested in its ability to recover different types of variables, including: (i) variables that are averages of sub-image properties, (ii) variables that not directly observable through daytime imagery but are a function of visible objects in the image, such as nighttime lights, and (iii) variables that are an underlying factor that determines what material appears in the image, such as elevation. Labels may also be a combinations of (i)-(iii), such as housing price or household income. An advantage of MOSAIKS is that it solves all these cases without any alteration of method. In the main text, we use the the same set of image features to predict all seven outcomes and, in principle, this set of features can be used to predict an unlimited number of outcomes (Note 2.3, so long as the

outcomes and the images are aligned as described in Note 2.2).

For each task, we obtain an up-to-date and geographically complete publicly available data-source to match with the images. Most of these data are based on measurements from 2010 - 2015, though our data on population density draws from sources that date back as far as 2005 in order to achieve global coverage. Our imagery data, from the Google Static Maps API (Note 1.2), was mostly acquired in 2018, though in some cases images may be a few years older.

**Task**	**Units**	**Native resolution**	**Data source**
Forest cover	% forest cover	$\sim$30m $\times$ 30m	(*2*)
Elevation	meters	$\sim$611.5m $\times$ 611.5m	(*37*)
Population density	people per sq. km.	$\sim$1km $\times$ 1km	(*38*)
Nighttime lights	nanoWatts/cm^2/sr	$\sim$500m $\times$ 500m	(*39*)
Income	USD per household	census block group	(*31*)
Road length	meters	polyline	(*40*)
Housing price	USD per sq. ft.	geocoded point data	(*41*)

Supplementary Table 1: **Data sources for all tasks.** Note that for all raster data sets (forest cover, elevation, population density, and nighttime lights) stated resolutions apply to grid cells located at the equator; raster size in Euclidean distance will vary with latitude.

1.1 Labels

Tasks were chosen to represent outcomes of classes (i)-(iii) above, subject to the condition that high resolution and up-to-date label data are available across the US. Below we describe these data sources. See Note 2.2 and Supplementary Figure 4 for a description of how we assign raw label data to images.

Forest cover To measure forest cover, we use globally comprehensive raster data from ref. (*2*), which is designed to accurately measure forest cover in 2010. This dataset is commonly used to measure forest cover when ground-based measurements are not available (*45, 46*). Forest in these data is defined as vegetation greater than 5m in height, and measurements of forest cover are given at a raw resolution of roughly 30m by 30m. These estimates of annual maximum forest

cover are derived from a model based on Landsat imagery captured during the growing season. Specifically, the authors train a pixel-level bagged decision tree using three types of features: "(i) reflectance values representing maximum, minimum and selected percentile values (10, 25, 50, 75 and 90% percentiles); (ii) mean reflectance values for observations between selected percentiles (for the max-10%, 10-25%, 25-50%, 50-75%, 75-90%, 90%-max, min-max, 10-90%, and 25-75% intervals); and (iii) slope of linear regression of band reflectance value versus image date." These estimates of forest cover were derived using different spectral bands than we observe in our imagery, and using information about how surface reflectance changes over the growing season, which we did not observe. This gives us confidence that we are indeed learning to map visual, static, high-resolution imagery to forest cover, rather than simply recovering the model used in ref. (*2*).[1]

Elevation We use data on elevation provided by Mapzen, and accessed via the Amazon Web Services (AWS) Terrain Tile service. These Mapzen terrain tiles provide global elevation coverage in raster format. The underlying data behind the Mapzen tiles comes from the Shuttle Radar Topography Mission (SRTM) at NASA's Jet Propulsion Laboratory (JPL), in addition to other open data projects.

These data can be accessed through AWS at different zoom levels, which range from 1 to 14 and, along with latitude, determine the resolution of the resulting raster. To align with the resolution of our satellite imagery, we use zoom level 8, which leads to a raw resolution of 611.5 meters at the equator.[2]

[1] These data were originally accessed at: `https://landcover.usgs.gov/glc/TreeCoverDescriptionAndDownloads.php`. They can now be found from the University of Maryland, Department of Geographical Sciences and USGS at `https://glad.umd.edu/dataset/global-2010-tree-cover-30-m`.

[2] We accessed these data via the R function `get_aws_terrain` from the `elevatr` package. Code and documentation can be found here: `https://www.github.com/jhollist/elevatr`.

The underlying data sources for the Mapzen tiles vary across locations in the following way:

- ArcticDEM terrain data DEM(s) were created from DigitalGlobe, Inc., imagery and funded under National Science Foundation awards 1043681, 1559691, and 1542736
- Australia terrain data © Commonwealth of Australia (Geoscience Australia) 2017
- Austria terrain data © offene Daten Österreichs – Digitales Geländemodell (DGM) Österreich
- Canada terrain data contains information licensed under the Open Government Licence – Canada
- Europe terrain data produced using Copernicus data and information funded by the European Union - EU-DEM layers
- Global ETOPO1 terrain data U.S. National Oceanic and Atmospheric Administration
- Mexico terrain data source: INEGI, Continental relief, 2016
- New Zealand terrain data Copyright 2011 Crown copyright (c) Land Information New Zealand and the New Zealand Government (All rights reserved)
- Norway terrain data © Kartverket
- United Kingdom terrain data © Environment Agency copyright and/or database right 2015. All rights reserved
- United States 3DEP (formerly NED) and global GMTED2010 and SRTM terrain data courtesy of the U.S. Geological Survey

Population density We use data on population density from the Gridded Population of the World (GPW) dataset (*38*). The GPW data estimates population on a global 30 arc-second

(roughly 1 km at the equator) grid using population census tables and geographic boundaries. It compiles, grids, and temporally extrapolates population data from 13.5 million administrative units. It draws primarily from the 2010 Population and Housing Censuses, which collected data between 2005 and 2014. GPW data in the US comes from the 2010 census.[3]

Nighttime lights We use luminosity data generated from nighttime satellite imagery, which is provided by the Earth Observations Group at the National Oceanic and Atmospheric Administration (NOAA) and the National Geogphysical Data Center (NGDC). The values we use are Version 1.3 annual composites representing the average radiance captured from satellite images taken at night by the Visible Infrared Imaging Radiometer Suite (VIIRS). We use values from 2015, the most recent annual composite available (*47*).

This composite is created after the Day/Night VIIRS band is filtered to remove the effects of stray light, lightening, lunar illumination, lights from aurora, fires, boats, and background light. Cloud cover is removed using the VIIRS Cloud Mask product. These values are provided across the globe from a latitude of 75N to 65S at a resolution of 15 arc-seconds. The radiance units are nW cm^{-2} sr^{-1} (nanowatts per square centimeter per steradian).

Like forest cover, these labels are themselves derived from satellite imagery. However, because they capture luminosity at night, while our satellite imagery is taken during the day, the labels for luminosity and the imagery used to predict luminosity represent independent data sources. Our ability to predict nighttime lights depends on how well objects visible during the day are indicative of light emissions at night.[4]

[3]These data can be accessed at http://sedac.ciesin.columbia.edu/data/collection/gpw-v4 and are licensed for use under a Creative Commons Attribution 4.0 International License.

[4]These data can be accessed at https://eogdata.mines.edu/products/vnl/, after registering for a free account.

Income We use the American Community Survey (ACS) 5-year estimates of median annual household income in 2015. These data are publicly available at the census block group level, of which there are 211,267 in the US, including Puerto Rico. On average, block groups are around 38 km^2, though block groups are smaller in more densely populated areas.[5]

Note that all estimates in this paper that are based off of the ACS use the Census Bureau Data API but are not endorsed or certified by the Census Bureau.

Road length We use road network data from the United States Geological Survey (USGS) National Transportation Dataset, which is based on TIGER/Line data provided by US Census Bureau in 2016. Shapefiles for each state provide the road locations and types, including highways, local neighborhood roads, rural roads, city streets, unpaved dirt trails, ramps, service drives, and private roads. Road types are indicated by a 5-digit code Feature Class Code which is assigned by the Census Bureau.[6] The variable we predict is road length (in meters), which is computed as the total length of all types of roads that are recorded in a given grid cell.

The Census Bureau database is created and corrected via a combination of partner supplied data, aerial images, and fieldwork. The spatial accuracy of linear features of roads and coordinates vary by source materials used. The accuracy also differs by region, causing cases in which some regions lack recordings of certain road types, the most common one being private roads and dirt trails. For example, private roads are rarely recorded in Indiana and some regions in Ohio (Supplementary Figure 1A), despite satellite images that suggest they are present (Supplementary Figure 1B).[7]

[5]These data are accessible using the `acs` package in R (*48*), table number B19013.

[6]`https://www.census.gov/geo/reference/mtfcc.html`.

[7]The data can be accessed at: `https://prd-tnm.s3.amazonaws.com/index.html?prefix=StagedProducts/Tran/Shape/`.

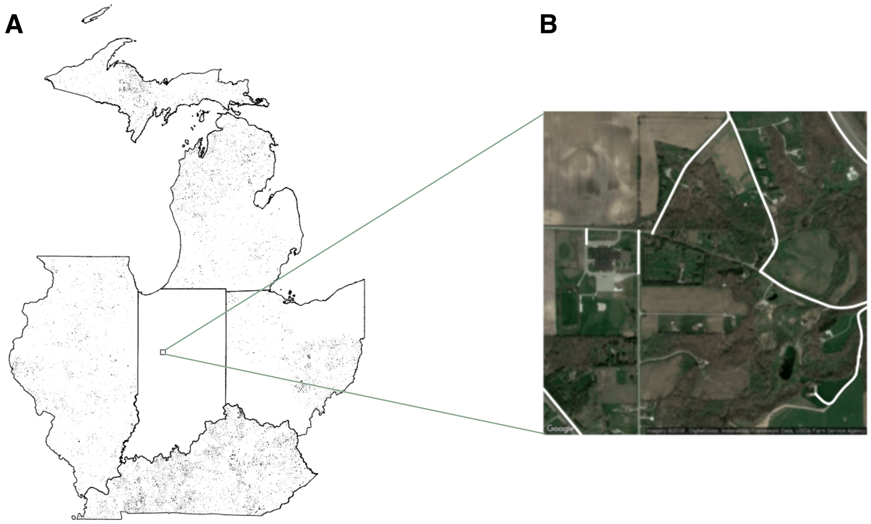

Supplementary Figure 1: **Quality of ground truth road data varies by region.** (A) Private roads in the northern Midwest recorded in the USGS National Transportation Dataset. The conspicuous lack of recorded private roads in Indiana and sections of Ohio suggests that road data quality in certain regions may be lacking. (B) Overlaying recorded roads of all types (shown in white) over a single satellite image (from Google Static Maps) in Indiana, demonstrates that some roads that are easily visible from satellite imagery are missing in the available data that we use to construct labels.

Housing price We estimate housing price per square foot using sale price and assessed square footage values for residential buildings. Data are provided by Zillow through the Zlllow Transaction and Assessment Dataset (ZTRAX). This dataset aggregates transaction and assessment data across the United States, combining reported values from states and counties with widely varying regulations and standards. Thus, significant data cleaning is required. Furthermore, because some states do not require mandatory disclosure of the sale price, we currently have limited data for the following states: Idaho, Indiana, Kansas, Mississippi, Missouri, Montana, New Mexico, North Dakota, South Dakota, Texas, Utah, and Wyoming. To address data quality issues, we develop a quality assurance and quality control (QA/QC) approach that is based on approaches employed in previous work (*49–51*) but adapted for our case.

ZTRAX contains data on the majority of buildings in the United States, initially comprising 374

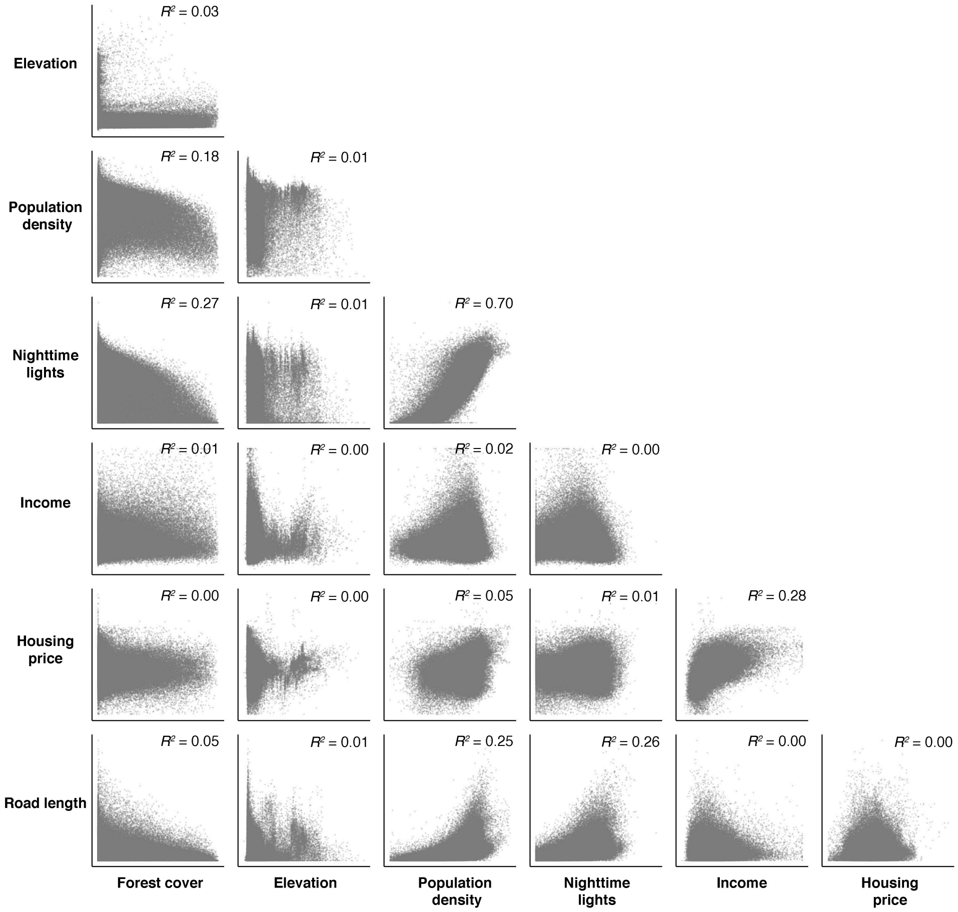

Supplementary Figure 2: **Correlation of labels across tasks.** Each figure shows a scatter plot of labeled outcomes for one of our seven tasks against another. All points come from a population-weighted random sampling of grid cells (as described in Note 2.1) across the US. Scatters and R^2 values are shown across approximately 100,000 grid cell labels, depending on the data availability for each task.

million detailed records of transactions across more than 2,750 counties. The data is organized into two components - *transaction data* and *assessment data*. These two datasets are linked, allowing us to merge the latest sale price of a property to the latest assessment data. To minimize the effect of nation-wide trends in housing price that would be unobservable from our cross-sectional satellite imagery, we limit our dataset to sales occurring in 2010 or later. Further, we restrict our analysis to buildings coded as "residential" or "residential income - multi-family"

and drop any sale that was coded as an intra-family transfer. To obtain a square footage value, we follow the example in Zillow Research's GitHub repository (*52*) and take the maximum reported square footage for a given improvement, and then sum over all improvements on a given property.

To reduce the number of potentially miscoded outliers at the bottom end of the distribution of sale price and property size, we drop any remaining sales that fall under $10,000 USD, any properties that fall under 100 sq. ft., and any $/sq. ft. values under $10. To address outliers on the high end of the distribution, we take this restricted sample and further cut our dataset at the 99th percentile of $/sq. ft. by state. Afterwards, we select the most recent recorded sale price for each property (divided by the most recent assessed square footage). We then average across all of the remaining units within each grid cell to comprise our final dataset of housing price per square foot.

To protect potentially identifiable information, our public data release contains housing price labels only for grid cells that contain 30 or more sales meeting the aforementioned criteria. This reduces the size of the dataset from $N = 80,420$ to $N = 52,355$ and makes the model performance obtainable by users better than that stated in the main text. For example, the public dataset will yield a test set R^2 of 0.60, rather than 0.52 (Supplementary Table 2). This could be due to the fact that the *average housing price* label we train on is noisier when estimated in a grid cell with few valid sales prices. It could also be because the *average housing price* of areas with few recent sales may be inherently harder to predict via satellite imagery than that of areas with a greater number of recent sales. Supplementary Figure 3 empirically demonstrates the performance effect of removing grid cells with few recent sales.

Correlation of outcomes across tasks The seven tasks described above were selected in order to evaluate the performance of MOSAIKS across many diverse contexts. Supplementary

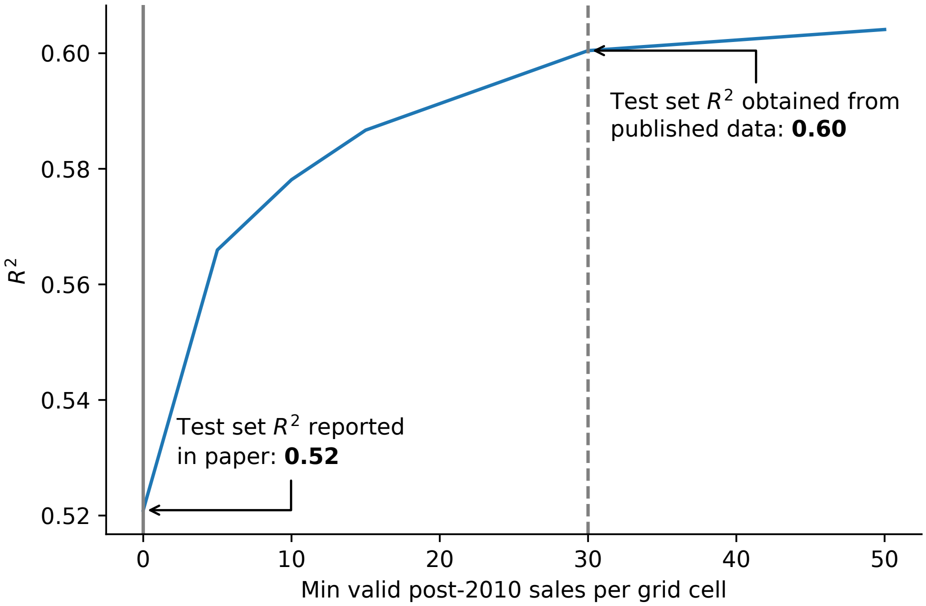

Supplementary Figure 3: **Test set performance when restricting the dataset of mean housing price labels**. Curve shows the R^2 obtained in the test set for the housing price task in our main prediction experiment (Supplementary Table 2), when removing data with low numbers of valid, recent sales of buildings within the associated grid cell. The dashed line indicates the restriction applied to the publicly available dataset.

Figure 2 evaluates the extent to which this was achieved, by plotting label values against one another. A few of the labels are moderately correlated, most notably population density and nighttime lights, but in general there is substantial orthogonal variation across these seven tasks.

1.2 Imagery

We use satellite imagery from Google Static Maps API (*36*), zoom level 16 (see Figure 1A for examples). This gives roughly 1km $\times$ 1km images which are 640 $\times$ 640 pixels across and 3 dimensions deep (red, green, and blue spectral bands). We coarsen these images to 256 $\times$ 256 $\times$ 3 prior to featurizing, meaning that our models are trained on images with roughly 4m resolution. These images can be composites of several satellite images – sources include the

Landsat, Sentinel, SPOT, Pleiades, WorldView and QuickBird satellites.[8] Prior to downloading, images were geo-rectified and pre-processed to remove cloud occlusions.[9]

2 Supplementary Note 2: Methods

This Supplementary Note describes the methods that we use to define samples (Note 2.1), to construct labels (Note 2.2), and to construct features (Note 2.3) for each image. It then describes how we separate data for training and evaluation (Note 2.4), train models (Note 2.5), test predictive skill (Note 2.6), test sensitivity to the dataset size (Note 2.7) and test model extrapolation performance (Note 2.8). Next, we describe tests of model performance at sub-label or "super" resolution as well as at the global scale (Notes 2.9 and 2.10).

2.1 Grid definition and sampling strategy

Grid definition: To evaluate the generalizability of MOSAIKS performance across tasks we need a standardized unit of observation to link raw labels for all tasks and imagery. To do this, we construct a single global grid onto which we project both satellite imagery and labeled data. We design the grid to match our source of satellite imagery to ensure adjacent images do not overlap. Each element of the grid, i.e. each "grid cell," was designed to be a square in physical space. Because the earth is a sphere, the angular extent of grid cells changes across latitudes.[10]

Sampling strategy: For our primary experiment in the continental US we subsample sets of 100,000 observations, roughly 1.25% of the grid cells in the continental US, using two dis-

[8] In some cases aerial photography is also integrated into images.

[9] More information is available at: `https://developers.google.com/maps/documentation/maps-static/dev-guide`.

[10] For the continental US (spanning 25 to 50 degrees latitude and -125 to -66 longitude), the grid cells are 0.0138 degrees in width (1.39 km) at the southern edge of the grid, and 0.0138 degrees in width (0.98 km) at the northern edge of the grid. The grid cells are 0.012 degrees in height (1.39 km) at the southern edge of the grid, and 0.0089 degrees in height (.98 km) at the northern edge of the grid.

tinct sampling strategies.[11] First, we sample uniformly-at-random (UAR) from all grid cells within the continental US. This sampling strategy is most appropriate for tasks like forest cover, where there is meaningful variation in most regions of the country. Second, we implement a population-weighted (POP) sampling strategy. To generate this sample, each grid cell is weighted by population density values taken from Version 4 of the Gridded Population of the World dataset, which provides a raster of population density estimates for the year 2015.[12] This weighted sampling strategy is most applicable to tasks like housing price, where the most meaningful variation lies in more populated regions of the US. We use the UAR grid when sampling population density to avoid any issues that might arise from sampling a task using the same variable as sampling weights. In both the UAR and POP samples, we randomly sample just once; all results in the paper are displayed using the same two subsets of the full grid. Note that these sub-sampled grid cells, by construction, are each covered by exactly one satellite image without having to process data over the entire US.

In our main results, we use the UAR sample for the forest cover, elevation, and population density tasks. We use the POP sample for nighttime lights, income, road length, and housing price. See Note 2.10 for a discussion of how we extend this grid and sampling procedure to the global scale.

2.2 Assigning labeled data to sampled imagery

To assign labels to each grid cell, we spatially overlay our raw labeled data and our custom grid. The native format and spatial resolution of the labeled data vary across the tasks studied, necessitating different aggregation or disaggregation procedures for each task. Here, we describe the

[11] We discard marine grid cells, but do not discard grid cells that are composed only of lakes or smaller inland bodies of water.

[12] These data are available at `http://sedac.ciesin.columbia.edu/data/collection/gpw-v4/sets/bro`

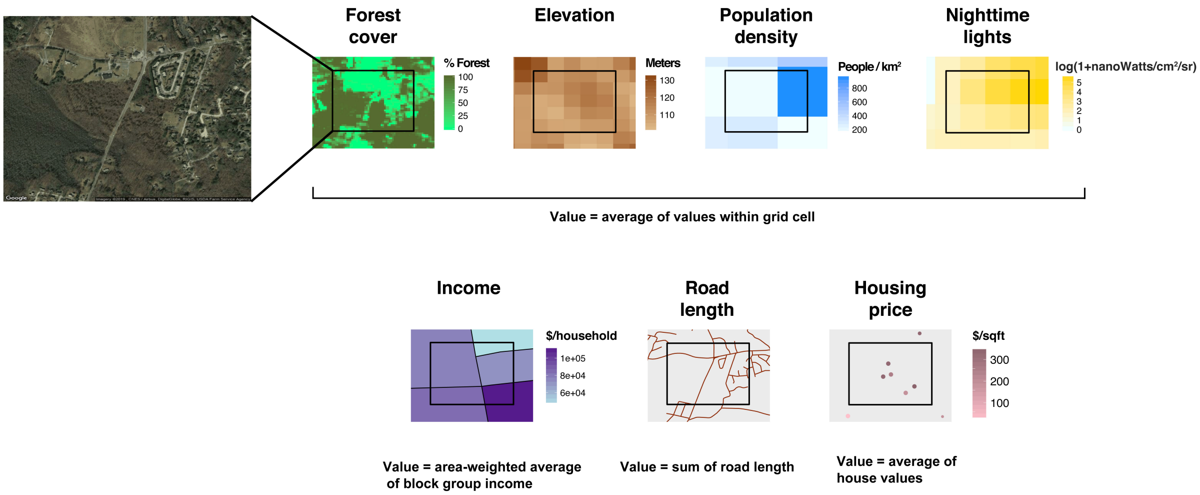

Supplementary Figure 4: **Calculation of grid cell labels from raw data.** We calculate labels by spatially overlaying our grid cells and raw labeled data. We calculate labels as the average of raw label values that fall within the grid cell, except for roads where we calculate the label as the sum of road length within the grid cell. Example image from Google Static Maps.

approach taken in each task (Supplementary Figure 4).

The raw forest cover, elevation, population density and nighttime lights data are provided natively as rasters with higher spatial resolution than our custom grid. For these tasks, we perform aggregation by calculating the mean of all labeled pixels with centroids that fall within the imagery grid cell. The resulting labels indicate mean forest cover, mean elevation, mean population density, and mean nighttime lights across the image grid cell.

Our road length data are provided as high-resolution spatial line segments. To aggregate these data to the image grid cell, we calculate the sum of road length segments within each image. The resulting labels indicate the total length of recorded roads that fall within an image grid cell.

Our housing price data are available as individual geocoded house sales. We aggregate these geocoded prices to the image grid cell by taking the average housing price per square foot across

all sale prices that fall within the extent of the image. The resulting labels indicate the average housing price per square foot across all observed houses within a grid cell.

Our income data are provided at the block-group level (see Note 1.1 for details). In some parts of the U.S., these block-groups are larger in total area than our image grid cells. However, in other regions, block-groups are smaller than our image grid cells. To treat both cases consistently, we aggregate incomes to the grid cell level by taking the weighted average of block-group incomes, where the weights are the area of intersection between the image grid cell and the block-group polygons. These weights are normalized to unity for each grid cell. The resulting labels indicate the area-weighted average median income across the grid cell.

Future users of a production-scale version of MOSAIKS would employ label data of arbitrary format and resolution. The above approaches provide guidelines for how to match various forms of label data to the pre-computed image feature grid, but other methods may be used. In the simplest case, for example, sparse point data could be directly matched to the nearest grid cell centroid.

2.3 Featurization of satellite imagery

Notation In our context, the input variable z is a set of satellite images $\mathbf{I}$, each corresponding to a physical location, ℓ. We use brackets to denote indexing into images, with colons denoting sub-regions of images (e.g. $\mathbf{I}_\ell[i,j]$ is the $(i,j)^{th}$ pixel of image $\mathbf{I}_\ell$, $\mathbf{I}_\ell[i:i+M, j:j+M]$ is the square sub-image of size $M \times M$ starting at pixel (i,j).) Because images have a third dimension (spectral bands), a colon $\mathbf{I}_\ell[i,j,:]$ denotes all bands at pixel (i,j). Indexing into non-image objects is denoted with subscripts (e.g. the kth element of vector $\mathbf{x}$ is denoted as $\mathbf{x}_k$ and the kth patch in a set of patches $\mathbf{P}$ is denoted as $\mathbf{P}_k$). We denote inner products with angular brackets $\langle \cdot, \cdot \rangle$ and the convolution operator with $*$.

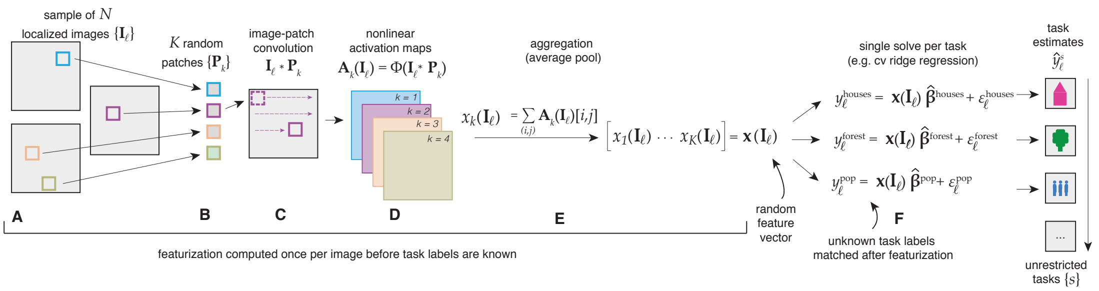

Supplementary Figure 5: **MOSAIKS process from featurization to multi-task prediction.** Given a large sample of N satellite images (A), a random sample of K patches (B) are drawn. (C) These K random patches P_k are convolved over each image $\mathbf{I}_\ell$ and (D) passed through a nonlinear function $\phi(\cdot) = \text{ReLU}(\cdot)$ to generate K activation maps. (E) Pixel-specific activations are pooled across each image to generate one set of $N \times K$ features that are stored and distributed to all users. (F) The same random feature vector x is used in cross-validated ridge regression across many distinct tasks, after labeled and geo-referenced data y_ℓ is matched to features from each image $\mathbf{I}_\ell$ (as shown in Figure 1B of the main text). (G) Models trained via ridge regression can be used to generate predictions across unrestricted tasks for any location with satellite imagery (icons indicate different tasks).

Connection to the kitchen sinks framework The *random kitchen sink* featurization used in MOSAIKS relies on a nonlinear mapping $g(z; \Theta_k)$, where z is an input variable and Θ_k is a randomly drawn vector. Here, we describe the implementation details of this featurization in the context of satellite imagery. Connecting our implementation and notation to the framework of random kitchen sinks, the random variables Θ_k are instantiated as the values of a random patch $\mathbf{P}_k$ and the bias b_k. The input variable z is an image $\mathbf{I}_\ell$, and $g(z; \Theta_k)$ represents the convolution of the patch over the image, followed by addition of the bias b_k and application of a element-wise ReLU function and an average pool, as described in the Methods of the main article and detailed below.

Methodological Details Supplementary Figure 5 depicts our featurization process. As described in Notes 1.1 and 2.1, we begin with two sets (uniform and population-weighted samples) of $N = 100,000$ satellite images, each of which measures $640 \times 640 \times 3$ pixels (the third di-

mension represents the visible red, green, and blue spectral bands). We then coarsen the images to $256 \times 256 \times 3$ pixels to reduce computation. Next, we draw $K/2 = 4,096$ small sub-image "patches" of size $M \times M \times 3$ uniformly at random from the 80,000 images that comprise our training and validation set, and calculate the negative of each patch to get another $4,096$ patches (Supplementary Figure 5A, 5B). Our chosen specification sets $M = 3$, so that each patch $\mathbf{P}_k$ is of dimension $3 \times 3 \times 3$ (see Supplementary Figure 6 for performance in experiments using different patch sizes).

We then "whiten" each patch by zero components analysis (ZCA), a common pre-processing routine in image processing (*53*). ZCA whitening pre-multiplies each patch by a transformation such that the resulting empirical covariance matrix of the whitened patches is the identity matrix. We then convolve each patch $\mathbf{P}_k$ over each of the N images (Supplementary Figure 5C) to obtain a set of $254 \times 254 \times 1$ pixel matrices for each image $\mathbf{I}_\ell$[13].During the convolutions each $3 \times 3 \times 3$ sub-image $\mathbf{I}_\ell[i : i + M, j : j + M, :]$ is also whitened according to the same whitening matrix as is applied to the patches.[14] We then apply a pixel-wise nonlinearity operator Φ to each resulting matrix to obtain K *nonlinear* activation maps $\mathbf{A}_k(\mathbf{I}_\ell) = \Phi(\mathbf{P}_k * \mathbf{I}_\ell + \mathbf{b}_k)$ for each image $\mathbf{I}_\ell$ (Supplementary Figure 5D) so that the $(i, j)^{th}$ pixel of the kth activation map is defined as

$$\mathbf{A}_k(\mathbf{I}_\ell)[i, j] = \Phi(\langle \mathbf{I}_\ell[i : i + M, j : j + M, :], \mathbf{P}_k \rangle + b_k), \quad (1)$$

where b_k is a bias term from the constant bias matrix $\mathbf{b}_k$, in which every element is equal to $b_k = 1$. We use $\Phi(\mathbf{I}_\ell; \mathbf{P}_k, \mathbf{b}_k) = \text{ReLU}(\mathbf{P}_k * \mathbf{I}_\ell + \mathbf{b}_k) := \max\{\mathbf{P}_k * \mathbf{I}_\ell + \mathbf{b}_k, 0\}$ as the nonlinear operator. We then aggregate across the image by taking the average of the nonlinear activation

[13]To improve efficiency of the featurization process, our implementation calculates the inner product of patch and image only for the original $K/2$ patches. We then create an additional $K/2$ values equal to the negative of each of the original inner products.

[14]In practice, we apply the whitening operator as a right multiplication to the original 8192×27 whitened patch matrix in order to reduce computation.

maps (Supplementary Figure 5E). The combination of the nonlinear operator $\Phi(\cdot)$ and average pooling composes the function $g(\cdot)$ above, and creates a scalar value for each patch k and image ℓ pair:

$$\mathbf{x}_k(\mathbf{I}_\ell) = \frac{1}{254^2} \sum_{i=1}^{254} \sum_{j=1}^{254} \mathbf{A}_k(\mathbf{I}_\ell)[i, j] \tag{2}$$

Stacking these scalars across all K patches provides the resulting K-dimensional feature vector, $\mathbf{x}(\mathbf{I}_\ell) := \begin{bmatrix} \mathbf{x}_1(\mathbf{I}_\ell) & \mathbf{x}_2(\mathbf{I}_\ell) & \dots & \mathbf{x}_K(\mathbf{I}_\ell) \end{bmatrix} \in \mathbb{R}^K$. This featurization thus embeds the original image $\mathbf{I}_\ell$ into a K-dimensional feature space, which can then be mapped to many different outcomes using task-specific models (s) implemented by researchers (r): $y_\ell^{s,r} = \mathbf{x}(\mathbf{I}_\ell)\boldsymbol{\beta}^{s,r} + \epsilon_\ell^{s,r}$, as illustrated in Supplementary Figure 5F. This linear relationship between labels and features may express a relationship between labels and image pixels that is highly nonlinear because the features themselves are nonlinear with respect to the images.

Patch size and sampling We approximate the idealized complete convolutional basis, which contains features for all patch sizes, with the simpler truncated basis where we use only a single patch size. Throughout our main analysis, we use a $3 \times 3 \times 3$ patch size for $\mathbf{P}_k$. While larger patches may, in principle, enable the detection of image features with larger spatial structure, we find that, in practice, patch size $M = 3$ performs best across all seven tasks (Supplementary Figure 6). This finding suggests that most information contained within satellite imagery of this resolution can be represented by local-level image structure, and that the inclusion of "non-local" relationships reduces the efficiency of the function approximator by introducing more degrees of freedom. This empirical finding is consistent with previous applications of kitchen sink features (*26*).

We draw patches randomly from the empirical distribution of $M \times M \times 3$ patches from our training data set of satellite images. Drawing patches from the empirical distribution, rather

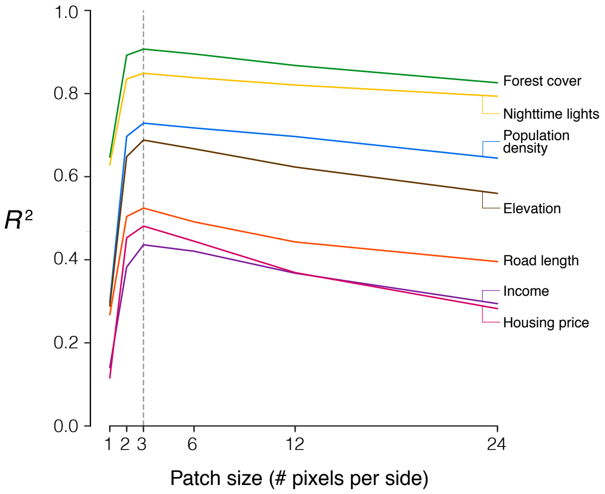

Supplementary Figure 6: **Performance by patch size.** Featurization in MOSAIKS relies on convolving an $M \times M \times 3$ patch $\mathbf{P}_k$ across satellite images. M indicates the width in pixels of each sub-image patch, and the third dimension indexes the 3 spectral bands used throughout the analysis in this paper (an analogous approach can be applied to hyperspectral data). This figure shows, for each task, test set R^2 for patch sizes $M = 1, 2, 3, 6, 12$ and 24, using $K = 2,048$ features for each M. The dotted gray line indicates the benchmark model used throughout the paper, with $M = 3$.

than generating them randomly, allows us to sample efficiently from the distribution of sub-images we will encounter in the sample. This patch selection process is almost identical to the filter selection methods described in refs. (*54–56*). It may be valuable for future research to explore whether MOSAIKS performance and computational efficiency could be improved through patch selection algorithms. For example, one goal in selecting patches-based features is to promote relative sparsity in the resulting patch-based features, as in ref. (*9*). However, any attempt to tailor patch selection or featurization to a particular task of interest requires sacrificing the generalizability of this task-agnostic featurization. It remains an open question whether a non-randomly selected set of basis patches could potentially achieve similar (or greater) performance than what we present here when applied to arbitrary new tasks.

Alternative interpretations relating MOSAIKS to kernels and CNNs The Methods section describes how MOSAIKS's convolutional random features enable nonparametric approximations of nonlinear functions through an embedding in a rich basis that expresses local spatial relationships. Here, we provide two alternative interpretations of the approach, the first relating to kernel methods and the second relating to convolutional neural networks. We believe these interpretations can provide useful lenses to consider why MOSAIKS works, and may also be helpful to researchers thinking about related problems.

First, one could interpret the design of MOSAIKS as if we were attempting to design a computationally tractable approximation to implementing a ridge regression using a convolutional kernel and the kernel trick. Under this interpretation, one could arrive at the same design of MOSAIKS using the following logic: (i) Design a kernel that allows us to describe the "similarity" of every image to every other image in the sample. (ii) For any new task, we want to use a kernel regression to predict the unobserved labels of new images based on their similarity to all other images—specifically, predicted labels would be a weighted sum of all observed labels using weights determined by this kernel-based measure of image similarity, i.e. the kernel trick. (iii) Unfortunately, calculating such a kernel exactly would be computationally intractable on a data set as large as the one we use, so instead use convolutional kitchen sinks (i.e. the featurization in MOSAIKS) to approximate the desired kernel regression. This last step follows from prior work demonstrating two concepts. First, random features can approximate the lifted feature space induced by well-known kernels (*57*) as the number of random features increases. Second, convolutions of random patches drawn from joint Gaussian distributions has been proven to approximate, in the limit, a kernel in which every sub-image from one image is compared with every sub-image from another using an arc-cosine distance function (*58*). Thus, convolutions with random patches should, in the the limit, approximate a kernel that compares every sub-image with every other sub-image in the sample. However, because our distribution

of patches is drawn from training imagery, rather than from Gaussian distributions, there is not an analytical expression that is known for the kernel being approximated by MOSAIKS in the limit.

The above logic would arrive at a design essentially the same as MOSAIKS, although it is not our preferred motivation or interpretation of why MOSAIKS works because it is a more complicated rationale than is needed. Ref. (*16*) showed that the existence of an associated kernel is not necessary for performance using kitchen sinks. Rather, it is simply the embedding of an input in a descriptive basis that provides the predictive skill, the insight that motivates our preferred—and we think simpler—interpretation presented in the main text. Nevertheless, the interpretation of MOSAIKS in the context of kernels motivates one way to understand the mechanism through which MOSAIKS achieves predictive skill at low computational cost. Namely, it enables the approximation of a nonparametric kernel regression, using some (unknown) fully convolutional kernel that is sufficiently rich to represent meaningful similarity between images but costly enough to prohibit a direct application of the kernel trick.

An additional way to contextualize MOSAIKS is in terms of its computational elements. In particular, MOSAIKS uses image convolutions and nonlinear activation operations common to convolutional neural networks (CNNs) (*59*). Indeed, MOSAIKS is mathematically identical to the architecture one would arrive at if one designed a very shallow and very wide CNN without using backpropogation and instead using random filters. Specifically, MOSAIKS could be viewed as a two-layer CNN that has an 8,192-neuron wide hidden layer with untrained weights that are randomly initialized by drawing from sub-images in the sample, and that uses an average-pool over the entire image. In contrast to the conventional CNN approach of optimizing weights (via backpropogation), the random initialization with no subsequent optimization significantly reduces training time and avoids numerical challenges associated with non-convex

optimization procedures (such as vanishing gradients). Thus, in the main text, we do not frame MOSAIKS as a CNN because MOSAIKS does not exploit the primary benefits of a deep CNN, since MOSAIKS lacks intermediate layers and does not implement backpropogation. Nonetheless, some readers may find this description more intuitive, and, as mentioned in the main article, we believe that the high performance of MOSAIKS might motivate the design of CNN architectures that share some of these computational elements.

Because deep CNNs are a state-of-the-art tool for SIML tasks, we provide further comparisons of MOSAIKS performance and cost relative to this benchmark in Notes 3.1 and 3.2, respectively.

2.4 Data separation practices and cross-validation

We split our data into a 20% holdout test sample and an 80% training and validation sample. Within the training and validation sample, we perform 5-fold cross validation in our primary analysis, splitting the training and validation sample into 5 sets of 80% training data (64% of full sample) and 20% validation data (16% of full sample), such that the validation sets are disjoint.

Creating the holdout test set Before any of the label data are touched, we remove a hold-out test set that is chosen uniformly at random from the entire sample, consisting of 20% of the original data. The analysis and diagnostic procedures that follow use only the remaining 80% of the observations. The held-out test set is only used once, for the purposes of comparison to the validation set performance in Supplementary Table 2. It is important to keep these data untouched until this point to ensure that our final performance results do not suffer from overfitting.

Tuning hyperparameters We choose the optimal λ in Eq. (4) for each outcome through 5-fold cross-validation over the training and validation sample. Specifically, λ is chosen to maximize average performance (R^2) across 5 folds, from a list of candidate values.[15] For tasks with the same sampling scheme (i.e. UAR versus population-weighted sampling), the folds are consistent across tasks, so that each of the five folds comprises the same set of locations across the tasks.

Using cross-validation to measure model robustness In addition to being a principled way of selecting hyperparameters, cross-validation gives us a notion of how robust our model is to changes in the training and validation samples. Since each of the 5 validation sets is disjoint and randomly selected, the empirical spread of performance across folds gives us a notion of the variability of our model when applied to new data sets from the same distribution. Understanding this variation is one way of understanding the performance of our model; it gives us a notion of variance of aggregated performance (e.g. R^2 over the entire sample, for a given set of hyperparameters). A useful aspect of MOSAIKS's low computational cost of model training, however, is that it enables researchers to calculate the variance of individual predictions by bootstrapping.

2.5 Training and testing the model

In our primary model (results shown in Figure 2 of the main text) we solve for grid cell labels as a linear function of random convolutional features using a ridge regression model and a cross-validation procedure. To obtain training and validation sets, we follow the data separation practices outlined in Note 2.4, and drop any observations with missing values. The resulting combined training and validation set sizes are N = 80,000 for forest cover, 80,000 for elevation,

[15] We choose these candidate values so as to ensure the chosen optimal λ is not the minimum or maximum of all λs supplied.

54,375 for population density, 80,000 for nighttime lights, 73,102 for income, 80,000 for road length, and 80,420 for housing price.

Population density, nighttime lights, and housing price have label distributions that are approximately log-normal (Supplementary Figure 7), so we take a log transformation of the labels. We add 1 before logging to avoid dropping labels with an initial value of zero (see Note 2.6 for performance in logs and levels for all tasks).[16]

With these labels and features in hand, we regress each outcome y_ℓ^s for each task s on features $\mathbf{x}_\ell$ as follows:

$$y_\ell^s = \mathbf{x}(\mathbf{I}_\ell)\boldsymbol{\beta}^s + \epsilon_\ell^s \tag{3}$$

We solve for $\boldsymbol{\beta}^s$ by minimizing the sum of squared errors plus an l_2 regularization term:

$$\min_{\boldsymbol{\beta}^s} \frac{1}{2}||y_\ell^s - \mathbf{x}(\mathbf{I}_\ell)\boldsymbol{\beta}^s||_2^2 + \frac{\lambda^s}{2}||\boldsymbol{\beta}^s||_2^2 \tag{4}$$

We use ridge regression across all outcomes to demonstrate the generalizability of using a single set of image features across many simple regression models. Further, this standardized methodology facilitates comparison of performance and sensitivity across tasks. We note that other modeling choices could potentially improve fit (e.g. using a hurdle model for zero-inflated outcome distributions such as road length); we leave such task-specific explorations for future research.

In visual display of results and calculation of performance metrics such as R^2, we clip our predictions for each task at the minimum and maximum values observed in the labeled data.

The resulting weights (i.e. regression coefficients) $\hat{\boldsymbol{\beta}}^s$ obtained from estimation of Eq. (3) indicate, along with the variance of the features, which features k (derived from random patch $\mathbf{P}_k$)

[16]Since housing price per square foot is always positive, for that variable we use just a log transformation.

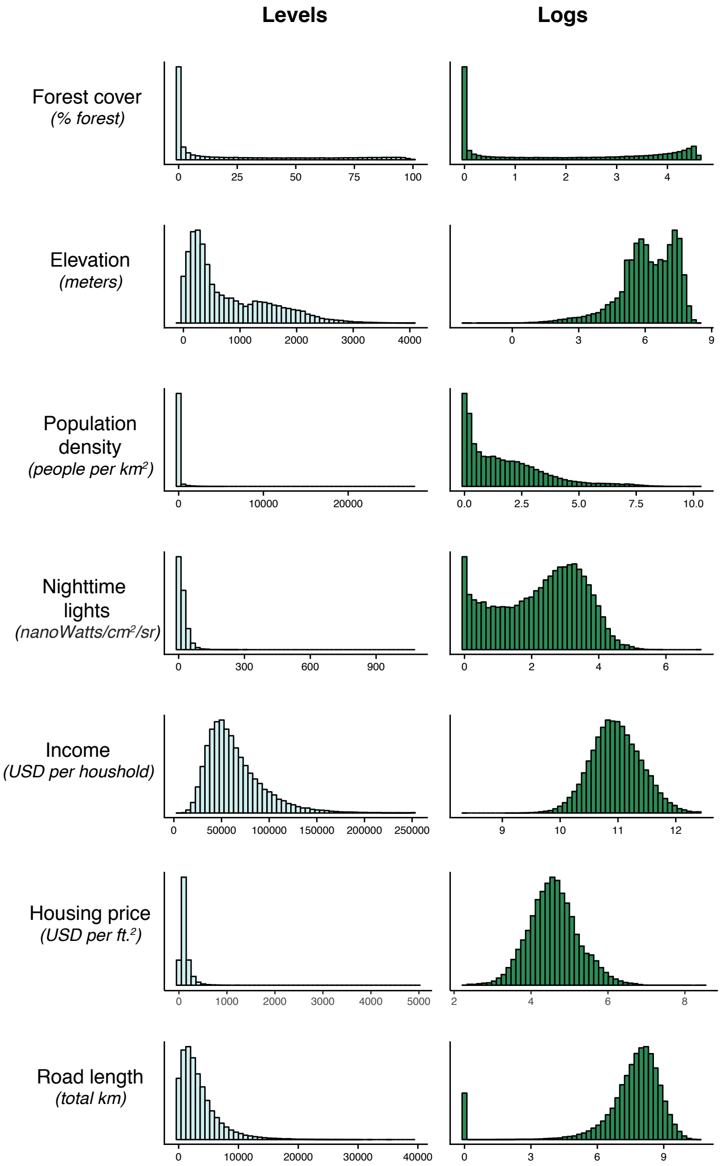

Supplementary Figure 7: **Distribution of outcome variables in levels and logs.** Histograms show the distribution of each outcome variable over all sampled image grid cells (approximately 100,000 observations, depending on data availability). Forest cover, elevation, and population density are sampled uniform at random across the continental US, while all other variables are randomly sampled with population weighting. The first column shows the distribution in levels, and the second in logs. For elevation, population density, nighttime lights, and road length, logs were taken after adding 1 to the raw values, given the propensity of zero values in these outcomes.

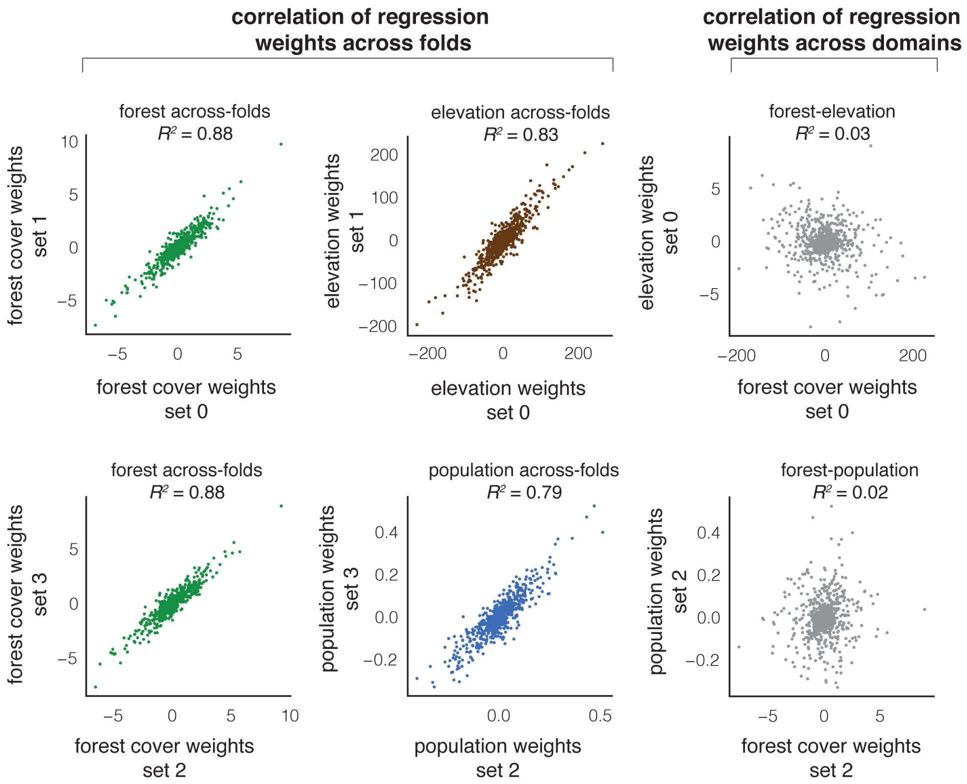

Supplementary Figure 8: **Regression weights across folds within a task vs. across tasks within a fold.** All scatterplots indicate regression weights for forest cover, elevation and/or population density. Each point depicts estimated coefficent values for the kth feature (β_k^s) when trained on either different samples or different labels. In the across-fold examples (first two columns), we learn weights for disjoint training and validation splits for the same task via cross-validation in which one fold acts as the training set and the other as the validation set. Values corresponding to each axis are the regression weights when that fold is the training set (e.g. the top left scatter shows $\{\beta_k^{forest1}, \beta_k^{forest0}\}$), and indicate a strong correlation across regression weights from different folds. In the across-task examples (last column), regression weights are shown for the same training and validation sets for two distinct tasks (e.g. the top right scatter shows $\{\beta_k^{elevation0}, \beta_k^{forest0}\}$). We see that there is virtually no correlation in regression weights across tasks, demonstrating that predictions across tasks lie in orthogonal subspaces of the feature space. Across all examples here, we set the number of random features to $K = 1,024$.

capture meaningful information for prediction in each task. Supplementary Figure 8 demonstrates that the recovered weights are stable across cross-validation folds within a task. The first two columns show standardized weights that are estimated from disjoint training and validation

splits for the same task.[17] Values corresponding to each axis are the regression weights estimated when the corresponding fold composes the training set. High R^2 values indicate a strong correlation between regression weights from *different folds within a single task* (forest cover, elevation, and population density are shown), demonstrating that similar linear combinations of features are selected by the regression model, even when the sample of training images changes. This suggests that specific sets of patches consistently contain valuable information in predicting outcomes for a specific task. However, different combinations of patches are useful for different tasks, and we find no correlation in the weights recovered *between tasks*. For example, in the last column of the figure, we show that regression weights that are recovered for forest cover and elevation (top right) are essentially orthogonal as are regression weights recovered for forest cover and population (lower right). In these two plots, regression weights are shown for the same training and validation sets, but for two distinct tasks. Sets of features that are relevant for prediction in one task appear to be irrelevant for another, as there is virtually no correlation in regression weights.

Intuition The consistency of weights recovered in MOSAIKS across folds within a task, and the orthogonality of weights recovered within a fold but across tasks, provides some intuition for why MOSAIKS provides consistent results and generalizes across a very large (potentially infite) number of potential tasks. The rich featurization $\mathbf{x}(\mathbf{I}_\ell)$ locates image $\mathbf{I}_\ell$ in a very high-dimensional (K-dimensional) feature space. Solving for $\boldsymbol{\beta}^s$ in Eq. (3) then identifies the K-dimensional vector $\boldsymbol{\beta}^s$ that points in the direction of most rapid ascent (the gradient vector) for labels y^s, when the position of images $\mathbf{x}(\mathbf{I}_\ell)$ are projected onto this vector. Because the feature space is so large — our baseline model has an 8,192-dimensional feature space — there

[17] For consistency across comparisons, R^2 is calculated on standardized regression weights, which have been demeaned and divided by their standard deviations. The number of random features is set to $K = 1,024$ for visual display purposes.

are a vast number of orthogonal gradient vectors that can be drawn through this space along which images can be organized for different tasks. The left and center panels of Supplementary Figure 8 illustrate that similar K-dimensional gradient vectors $\boldsymbol{\beta}^s$ are selected when solving for the same task but using different samples (each point depicts an element of the vector $\boldsymbol{\beta}^s$). The right panels shows that for different tasks, the gradient vectors are orthogonal and point in completely unrelated directions in the feature space. This orthogonality means that predictions $\hat{y}^s$ for different tasks will be independent of one another, even though both are constructed as linear combinations of the same set of features.

2.6 Primary model test set performance, robustness to functional form, and spatial distribution of errors

Here, we describe how we test for overfitting to the training and validation set in our primary model, test for primary model performance robustness to alternative functional forms, and characterize the spatial distribution of primary model error.

Performance in a holdout test set To test for overfitting, we evaluate the performance of our primary model on a randomly sampled 20% holdout set. These data were never used for model selection and were only touched at the end of our analysis to check for overfitting. To conduct this test, for each outcome, we use cross-validation within the training set to determine the outcome-specific optimal λ. We then retrain the model on the full training set using this optimal λ, and evaluate this model on the holdout test set. We find that performance in the test set is nearly identical to that of the validation set (Supplementary Table 2), which indicates that our models were not overfit to the data. For some performance metrics, such as the maps in the main text, we present validation set performance (instead of the test set) because the sample is larger and the performance is unchanged.

Task	Cross-validation R^2	Test set R^2
Forest cover	0.91	0.91
Elevation	0.68	0.68
Population density	0.73	0.72
Nighttime lights	0.85	0.85
Income	0.45	0.45
Road length	0.52	0.53
Housing price	0.50	0.52

Supplementary Table 2: **Model performance in the hold out test set.** For each outcome, we use 5-fold cross-validation within the training/validation set using 80% of our labeled data to optimally select task-specific hyperparameters in ridge regression (i.e. λ). We then retrain each model on the full training set using this optimal λ. Performance on the validation set (column 1) is compared to that of the held out test set (column 2).

Robustness of model to alternative functional forms Throughout the main text, we report primary model performance in each task from a model estimated with labels that are either logged (e.g. population density), or in levels (e.g. forest cover). The decision regarding functional form for each task was made based on the underlying distribution of labels across our image grid cells. Many outcomes, such as housing prices, display exceptionally skewed distributions that approximate log-normality (see Supplementary Figure 7). For these outcomes, we take the natural log of the image grid cell values in model training and testing. Supplementary Table 3 shows model performance for all tasks under both the levels and logs functional forms.[18] Tasks with highly skewed distributions, such as population density, housing price per square foot, and nighttime lights have substantially higher performance (R^2 increases by 10-64%) after being logged. Tasks whose labels display much less skew in levels, such as road length, income, and elevation show small to modestly reduced performance (4-21%) when their outcomes are modeled in logs.

[18]In tasks where negative values or zeros are present (e.g. forest cover, elevation, and nighttime lights), we drop negative values and add one to zero values before taking logs for this test.

Task	Log model R^2	Levels model R^2
Forest cover	0.90	**0.91**
Elevation	0.58	**0.68**
Population density	**0.73**	0.56
Nighttime lights	**0.85**	0.77
Income	0.43	**0.45**
Road length	0.41	**0.52**
Housing price	**0.50**	0.44

Supplementary Table 3: **Model performance across tasks and functional forms.** All R^2 values indicate performance using the optimal hyperparameter λ after 5-fold cross-validation. In the log model, the outcome variable is defined as the natural logarithm of the original labeled data (e.g. natural log of the average forest cover over an image gridcell). In the levels model, the outcome variable is simply the level of the aggregated labeled data, as defined in Note 2.2. Values in bold are reported in the main text.

Spatial distribution of errors Supplementary Figure 9 shows the distribution of errors over space, for the model predictions presented in Figure 2. The model systematically over-predicts low values and under-predicts high values across all tasks. This is likely due to our choice of ridge regression, which favors predictions that tend toward the mean due to the ℓ_2 penalty. The structured correlation of errors across space suggests that there is substantial room for model improvement, potentially from including task specific knowledge. For example, our models of housing price and elevation could likely, respectively, be improved by adding in information about school districts –to address clustering of house price error in parts of big cities – or location – to help identify large areas of high elevation such as the Rocky Mountains. We recognize that there exists substantial room for task-specific model performance, which we leave for future research. Further, discontinuities in the error structure over political boundaries can help identify inconsistency in label quality. For example, the sharp increase in road length prediction error moving across the border from Louisiana to Texas suggests that the raw data labeling in these two states may differ methodologically, which introduces error into the label,

and in turn, the model.

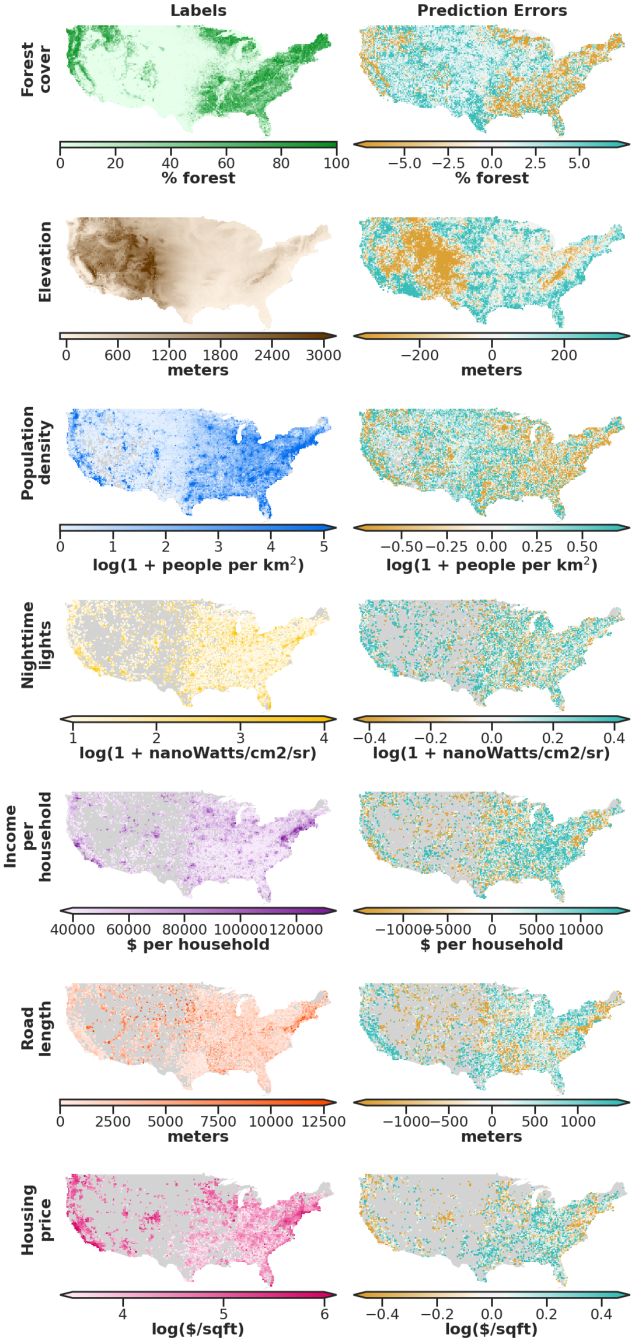

Supplementary Figure 9: **Labels and prediction errors over space for each task.** Left maps: ∼80,000 observations used for training and validation, aggregated up to 20km x 20km cells for display (precise number of observations varies by task based on data availability; see Note 2.5). Right maps: prediction errors from concatenated validation set estimates from 5-fold cross-validation for the same ∼80,000 grid cells, identically aggregated for display.

2.7 Altering the number of features and training set size

To better understand factors that could improve the primary model, we test the sensitivity of its performance to the number of features and the training set size (results shown in Figure 3 in the main text). Understanding the returns to additional features and observations enables better optimization of model performance given cost constraints.

Since features in MOSAIKS are generated randomly, there is no theoretical reason to select a specific number of features. To test the sensitivity of the primary model performance to the number of features, we train a model identically to our primary specification (Note 2.5) except that we vary the number of features across the values $\{100, 200, 500, 1000, 2000, 4096, 8192\}$ (Figure 3A). For each set of features and each task, we conduct 5-fold cross-validation to recover the optimal hyperparameter λ.

Notably, using only 100 features recovers a substantial amount of the variation across tasks. Of the tasks, the least variation is recovered for income (R^2 using 100 features is 81% of R^2 using 8,192 features) and the most variation is retained in nighttime lights (R^2 using 100 features is 96% of R^2 using 8,192 features). This suggests that in computation or memory-limited settings, fewer features could be used with only minor losses in performance. On the other hand, even with 8,192 features, performance does not fully flatten out (on a logarithmic scale). This suggests that performance could be improved further by increasing the number of features past $K = 8,192$. At the limit of our testing, a doubling of K from $4,096$ to $8,192$ led to a largest performance increase of 0.026 R^2 for income and a smallest of 0.010 R^2 for forest cover.

To test the sensitivity of primary model performance to the number of training samples, we train a model identical to our primary specification (with 8,192 features) except with a varying

size of training set (from 500 to 64,000 images) (Figure 3A).[19] In cases where the training set has fewer than 64,000 total observations due to missing data (e.g. population density, income, road length and housing price), we use the full training data set to construct our largest training sample.

Similarly to increasing the number of features, increasing the training set size increases model performance with diminishing marginal returns. Notably, models trained on only 500 observations recover at minimum 56% (road length) of performance relative to $N = 64,000$ and at maximum 87% (forest cover), excluding income and housing price, which require larger samples to attain performance. This suggests that, for all but the most difficult SIML tasks, MOSAIKS may be useful even when label collection is very costly. For the tasks with the best R^2 performance (forest cover, nighttime lights), performance plateaus out as the number of training observations approaches $64,000$. However, for the remaining five tasks, these results show that more training data could substantially increase performance further. The range of performance gain from increasing $N = 32,000$ to $64,000$ is bounded below by forest cover ($.005\ R^2$) and above by road length ($.027\ R^2$).

2.8 Testing generalizability across space and comparison to kernel-based interpolation.

To understand the ability of our model to predict outcomes in large contiguous regions with no ground truth, we design an experiment where we evaluate models using training and validation sets that are increasingly far away from each other in space. Specifically, we iteratively create a grid over the US with a side length of δ degrees and then use this grid to divide the training and validation dataset ($N = 80,000$) into spatially disjoint sets of roughly equal size. We create these disjoint sets by assigning observations that lie in every other box within the grid to the

[19]The same per-fold validation sets are used for each iteration of this analysis as well as for the primary analysis and for the test of model performance sensitivity to the number of features.

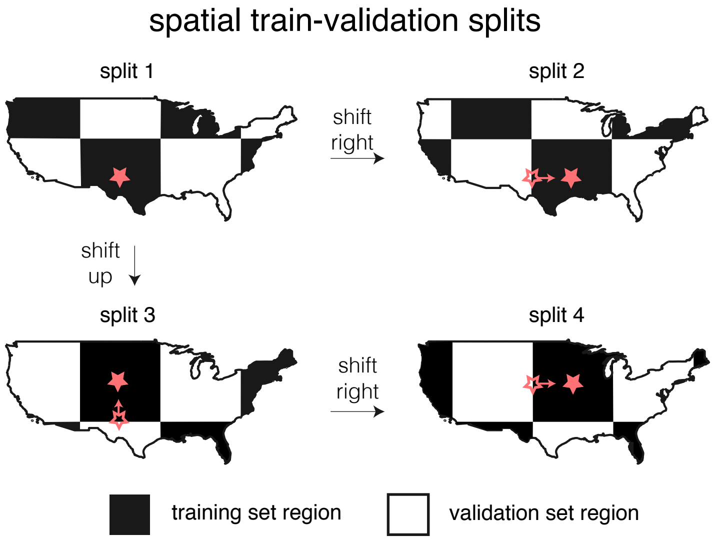

Supplementary Figure 10: **Illustration of the procedure to systematically shift train and validation sets in space when assessing the performance of MOSAIKSover regions with no ground-truth data.** To assess the ability of MOSAIKS to generate meaningful predictions when extrapolating across large spatial distances, we conduct a "checkerboard" experiment (Note 2.8, Figure 3B-C of the main text) in which the training set ("black squares") and validation set ("white squares") are separated by increasingly large distances. The length of a square in each experiment is δ, measured in degrees. This figure demonstrates the four different train/validation splits that are created by shifting a given spatial checkerboard (split 1) by $\delta/2$ to the right (split 2), $\delta/2$ up (split 3), and both simultaneously (split 4).

train set and test set, respectively, creating a checkerboard pattern with the train set and test set, as shown in Figure 3B. We vary the width δ of each square in the grid range across the values of $\{0.5, 1.5, 2, 4, 6, 8, 10, 12, 14, 16\}$ degrees (roughly 40 to 1400 km) in sequential runs of the experiment. As δ increases, validation set observations become on average farther away from the training set points. This distance makes prediction on the validation set more difficult, because observations in the validation set are now likely to be less similar to those in the training set. We learn the model on the training set using ridge regression. To assess the stability of this performance, we offset the checkerboard and re-run the above analysis four times – once in the original location and then three more times – shifting the grid up, right, and both up and right

by half the width of the grid (see Supplementary Figure 10). The ℓ_2 regularization term, λ, is selected to maximize average performance in the four validation sets, as we would select in in a standard cross-validation procedure.

The performance plotted in Figure 3C is the performance on the the resulting validation sets. We find that across most tasks, performance degrades only slightly as the distance between training observations and testing observations increases. This suggests that MOSAIKS is indeed learning image-label mappings that transfer across spatial regions.

Comparison of MOSAIKS to kernel-based spatial interpolation In these experiments we demonstrate that MOSAIKS outperforms spatial interpolation (or extrapolation, depending on geometry) – a commonly used simple technique to fill in missing data (Figure 3C). This suggests that MOSAIKS, and SIML generally, exploits the spectral and structural content of information within an image to generate predictions at national scale that extend beyond what can be captured by geographic location alone.

We compare MOSAIKSto kernel-based spatial interpolation using a Gaussian Radial Basis Function (RBF) kernel, a simple and general widely used approach. In this approach, the value for a point in the validation set at location $\ell_v \in \mathbb{R}^2$ is predicted to be a weighted sum of the values of all the points in the training set ℓ_t, as follows:

$$\hat{y}_v^s = \frac{\sum\limits_{\ell_t \in [\text{Train}]} y_t^s w(\ell_t, \ell_v)}{\sum\limits_{\ell_t \in [\text{Train}]} w(\ell_t, \ell_v)}; \qquad w(\ell_t, \ell_v) = e^{-\frac{1}{2\sigma^2}\|\ell_t - \ell_v\|^2} \tag{5}$$

Here, w is the weight assigned to each observation in the training set based on kernel values that are indexed to distance, such that w decreases as the distance between the point being predicted and the point in the training set increases. We select σ – the parameter that determines the

rate at which w degrades with distance – to maximize average performance on the validation set across all four spatially-offset runs, similar to how we tune λ in the spatial extrapolation experiment described above. The optimal value of the bandwidth parameter σ will depend on the task at hand, as well as the average distance from points in the validation set to points in the training set. To ensure comparability, spatial interpolation based predictions and performance are computed for the exact same samples as used for MOSAIKS in each checkerboard partition.

2.9 Label super-resolution

As discussed in the methods summary, the featurization method in MOSAIKS exploits the fact that many image-level outcomes of interest are linearly decomposable across sub-image regions. This is done by creating image-level features that are averages of statistics from all sub-image regions. Because these features are ultimately used in linear regression, a natural property of this approach is that weights estimated in this linear regression can be used not only to generate predictions of outcome variables at the image-scale, but also at the scale of any sub-image region. As satellite imagery are available at increasingly high spatial resolution, this "label super-resolution" property is both practical and powerful, enabling researchers to generate novel predictions at higher resolution than available ground truth data.

This section gives mathematical justification for a simple method to use MOSAIKS to predict outcomes of interest at a finer resolution than available labeled data. We display the label super-resolution properties of MOSAIKS visually, and quantitatively document the empirical performance of this label super-resolution approach.

Why MOSAIKS naturally achieves super-resolution for label predictions Given an image-label pair $\{\mathbf{I}_\ell, y_\ell^s\}$, the goal of label super-resolution is to resolve which sub-regions of the image $\mathbf{I}_\ell$ contribute to high or low values of y_ℓ^s. Recall that for image $\mathbf{I}_\ell$, feature vector $\mathbf{x}(\mathbf{I}_\ell)$ is a K dimensional vector, where each scalar element $\mathbf{x}_k(\mathbf{I}_\ell)$ of $\mathbf{x}(\mathbf{I}_\ell)$ is an average across the pixels

of the image of the values obtained by convolving sub-regions of the image with patch $\mathbf{P}_k$. As in Note 2.3, denote by $\mathbf{X}$ the full random feature matrix in $\mathbb{R}^{N\times K}$, so that $\mathbf{X}_{\ell k}$ denotes the kth element of the feature vector describing image $\mathbf{I}_\ell$. By Eq. (2), we can decompose the feature elements as:

$$\mathbf{X}_{\ell k} := \mathbf{x}_k(\mathbf{I}_\ell) = \frac{1}{254^2}\sum_{i=1}^{254}\sum_{j=1}^{254}\mathbf{A}_k(\mathbf{I}_\ell)[i,j] \tag{6}$$

where $\mathbf{A}_k$ is the activation map associated with patch $\mathbf{P}_k$. Since we are using a linear model to form predicted values, we can trace these values back to subregions of the original image. When we perform a linear regression for task s, the resulting regression weights are a vector $\hat{\boldsymbol{\beta}}^s \in \mathbb{R}^K$ such that the scalar $\hat{\beta}^s_k$ describes the relative weight of feature k in the image-scale predictions. The prediction of outcome s using image $\mathbf{I}_\ell$ thus decomposes as:

$$\begin{aligned}
\hat{y}^s_\ell &= \mathbf{X}_\ell \hat{\boldsymbol{\beta}}^s &(7)\\
&= \sum_{k=1}^{K}\mathbf{X}_{\ell k}\cdot\hat{\beta}^s_k &(8)\\
&= \sum_{k=1}^{K}\left(\frac{1}{254^2}\sum_{i=1}^{254}\sum_{j=1}^{254}\mathbf{A}_k(\mathbf{I}_\ell)[i,j]\right)\cdot\hat{\beta}^s_k &(9)\\
&= \frac{1}{254^2}\sum_{i=1}^{254}\sum_{j=1}^{254}\underbrace{\left(\sum_{k=1}^{K}\hat{\beta}^s_k\cdot(\mathbf{A}_k(\mathbf{I}_\ell)[i,j])\right)}_{\text{super-resolution prediction}} &(10)
\end{aligned}$$

where the third line follows from substituting $\mathbf{X}_{\ell k}$ according to Eq. (2). Therefore, we can associate with each pixel indexed by (i,j) a predicted super-resolution value:

$$\hat{y}^s_{\ell,(i,j)} = \sum_{k=1}^{K}\hat{\beta}^s_k\cdot(\mathbf{A}_k(\mathbf{I}_\ell)[i,j]) \tag{11}$$

which is that pixel's predicted label value, and thus its contribution to the overall predicted image-level label value $\hat{y}_\ell$ for $\mathbf{I}_\ell$. We use a Gaussian filter to smooth these per-pixel predictions to enforce spatial consistency and reduce variance of the high-resolution predictions, using

a kernel bandwidth of $\sigma = 16$ pixels. These smoothed pixel-level predictions can be average-pooled to larger sub-image scales as shown in Figure 4C. The procedure to construct label super-resolution predictions, and a comparison to the procedure to construct image-level predictions, is illustrated in Supplementary Figure 11.

Supplementary Figure 12 demonstrates empirical performance of Eq. (11) using ten examples of this approach at label super-resolutions on both the forest cover and population density outcomes. The ten images were randomly selected from the union of observations with forest cover $> 10\%$ and population density > 100 people/km^2 to ensure that all images considered had a non-negligible value for each variable.[20]

In our formulation, super-resolution label predictions are easily estimable during featurization. Consider again the per-pixel contributions of Eq. (11). An alternative way to express this is

$$\hat{y}^s_{\ell,(i,j)} = \left(\sum_{k=1}^{K} \hat{\beta}^s_k \cdot \mathbf{A}_k(\mathbf{I}_\ell) \right) [i,j] \tag{12}$$

That is, label super-resolution estimates are just a linear combination of the activation maps $\mathbf{A}_k(\mathbf{I}_\ell)$ weighted by $\hat{\beta}^s_k$ (see Supplementary Figure 11). Every time we featurize a new image $\mathbf{I}'_\ell$, we must perform the step of computing the K activation maps $\{\mathbf{A}_k(\mathbf{I}'_\ell)\}_{k=1}^K$ (Supplementary Figure 5D). Therefore, if we already have a suitable regression weight vector $\hat{\boldsymbol{\beta}}^s$ for task s, for any new images $\mathbf{I}'_\ell$ that we featurize, we can compute the label super-resolution predictions $\sum_{k=1}^K \hat{\beta}^s_k \cdot \mathbf{A}_k(\mathbf{I}_\ell)$ as weighted combinations of the activation maps at negligible additional cost, prior to pooling, in the existing featurization pipeline.

[20] To ensure that weights decomposed as a sum, as in Eq. (11), we used level values (i.e. not log-transformed) for population density labels in Supplementary Figure 12.

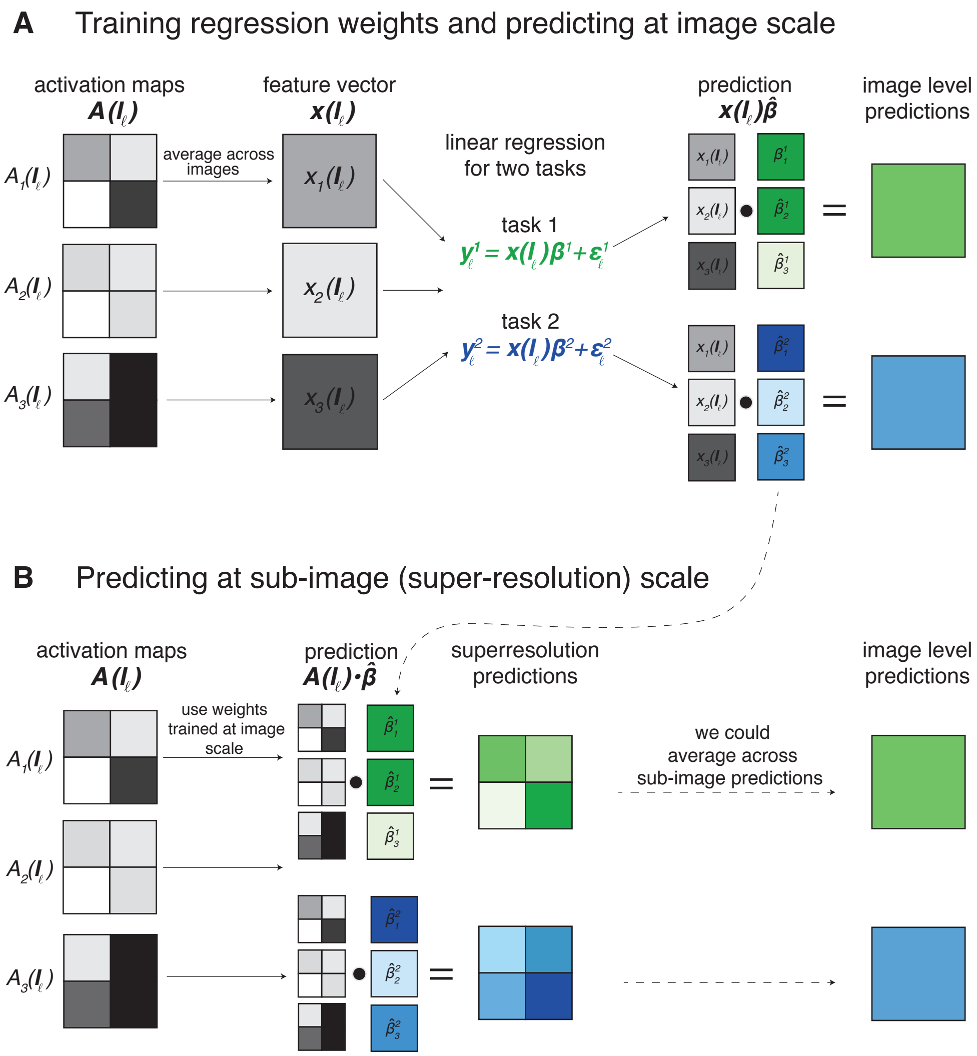

Supplementary Figure 11: **Illustration of the procedure to construct predictions at image resolution and label super-resolution.** Panel A illustrates the standard MOSAIKS prediction pipeline. After convolution with random patches, nonlinear activation maps $\mathbf{A}_k(\mathbf{I}_\ell)$ are averaged across images to construct a set of image-level features $\mathbf{x}_k(\mathbf{I}_\ell)$ used in linear regression to generate predictions at image-scale (Note 2.3). Panel B illustrates how the weights trained using labels and features at image-scale in panel A can be used to generate predictions at resolutions higher than the images and labeled data, achieving predictions at label super-resolution. The scalar product of the entire activation map $\mathbf{A}_k(\mathbf{I}_\ell)$ and the estimated weights vector $\hat{\boldsymbol{\beta}}$ generates label super-resolution predictions at any desired sub-image scale larger than pixel-level. The last column of panel B illustrates the fact that label super-resolution predictions, when averaged across an image, are identical to predictions generated from the standard process in panel A.

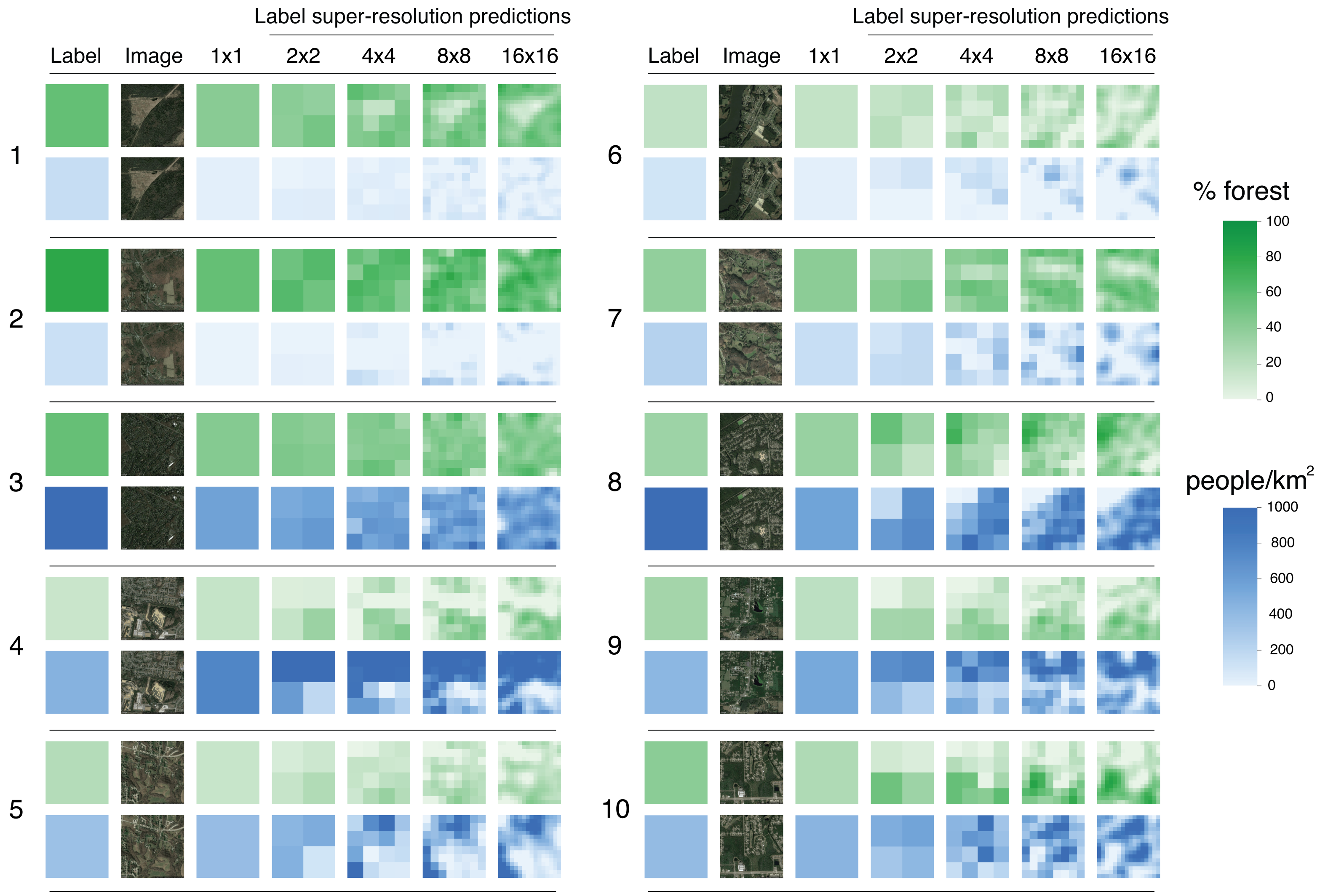

Supplementary Figure 12: **Label super-resolution performance across ten randomly selected images.** Each set of images indicate the image-level labels (column 1), the image itself from Google Static Maps (column 2) and predicted outcomes from MOSAIKS at increasing levels of label super-resolution (columns 3-7). These ten examples were selected uniformly at random from images in which our labels indicated at least 10% total forest cover and at least 100 people/km^2.

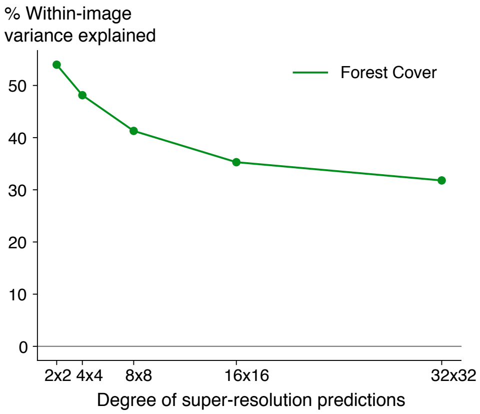

Supplementary Figure 13: **Systematic evaluation of *within-image* R^2 recovered in the forest cover task.**

Evaluating label super-resolution performance

To systematically evaluate the ability of MOSAIKS to accurately predict outcome labels at super-resolution, we evaluate the within-image label variation that MOSAIKS's label super-resolution predictions accurately explain. We use forest cover for this test because the raw label resolution is substantially finer than the grid cell used to construct labels (see Note 2.1 and Supplementary Figure 4), so we are able to attach "true" labels to super-resolution predictions within each image. In our main analysis, we construct grid cell forest cover labels by averaging fine-resolution raw forest cover data (see Note 2.2). Here we leverage the fine resolution of the raw data to compare label super-resolution performance of a model trained on aggregated labels but tested on high-resolution raw forest cover data.

Specifically, we learn regression weights $\hat{\boldsymbol{\beta}}^s$ using a ridge regression applied to image-level labels from the full U.S. UAR sample ($N = 100,000$). We do so for multiple regularization parameters, λ, then make label super-resolution predictions on a "validation" set of 1,000 images.[21] We use the R2 score for 32x downscaled predictions (32x32 predictions per image) to

[21]Note that none of the pixel-level values in this validation set are used in the ridge regression, but the corresponding image-level labels are in-sample.

choose optimal values for λ and σ (the Gaussian filter length scale).[22] Next, we use the weights derived from our image-level ridge regression, along with the corresponding optimal λ and σ, to make label super-resolution predictions for 16,000 additional images drawn randomly from the full set of 100,000 (excluding the 1,000 used for choosing hyperparameters). Lastly, we aggregate these pixel-level predictions to coarser sub-image scales, where increasing aggregation (lower label super-resolution factor) reduces noise in the predictions at the cost of lower resolution.

We assess the performance of label super-resolution at a variety of scales by calculating the percent of the variance of the raw within-image forest cover labels that can be explained by the super-resolution label predictions at each scale. For example, to assess the performance of 2×2 label super-resolution predictions, we average predictions from the 254×254 label super-resolution predictions by quadrants, resulting in four predicted values (twice the original resolution).[23] We perform the same per-quadrant average for the raw fine-resolution forest cover labels. We demean both the within-image predictions and labels to eliminate across-image variation, thereby focusing this test on the ability of the predictions to explain residual within-image variation. We then concatenate these within-image predictions and labels across the $N = 16,000$ images, so that the resulting R^2 value reported is the percent of super-resolution label variance explained by label super-resolution predictions, across $64,000 = 16,000 \cdot 2^2$ label-prediction pairs.

The resulting performance of label super-resolution predictions at different scales is shown in Supplementary Figure 13 for width scales of 2×2, 4×4, 8×8, 16×16, and 32×32. We test up to $w = 32$ because the native width of the forest cover labels ($\sim$ 30m) is just under $1/32$

[22] The optimal $\lambda = 1e5$ is higher than that chosen to optimize image-level predictions (Figure 2), likely due to increased noise in sub-image predictions.

[23] For the analysis, we clip the images and predictions to 224 x 224 pixels so they are evenly divisible by a 32x super-resolution factor.

the width of the original image ($\sim$ 1km). Label super-resolution predictions are trained only on the aggregate label at the image-level. Nonetheless, as Supplementary Figure 13 shows, we are able to explain over 50% of the within-image label variations at 2×2 super-resolution, and over 30% of the variation using 32×32 super-resolution grids.

Comparisons to other within-image prediction algorithms The derivation leading to Eq. (11) has a very similar form to the derivation of class activation mapping in (*60*). Similar to our goal of label super-resolution, class activation mapping identifies image sub-regions that contribute to the overall prediction for that image. Class activation mapping usually refers to finding discriminative regions of an image that help explain a binary classification decision; we differ from this in our objective of predicting regression values at finer-resolution than the image-sized labels. We use the term "label super-resolution" (also used in (*34*)) to further distinguish our approach from *image* super-resolution methods in image processing and microscopy, which increase the resolution of the image itself, rather than the associated labels.

A approach to MOSAIKS's label super-resolution predictions are methods specifically designed for pixel-level classification, or *semantic labelling* of satellite imagery (*61, 62*). However, these approaches make use of sub-image labels for training, as opposed to our setting, where only one label per image (per task) is provided. For example, (*34*) studies the case of weakly supervised image segmentation, predicting land cover at finer resolution than the provided labels, which are already at sub-image resolution. Some such semantic labelling approaches use a downsample-then-upsample approach inspired by auto-encoders (*63*) to learn lower-dimensional latent representations which are then up sampled to image-size prediction maps from which per-pixel classifications can be made. The upsampling procedure introduces more parameters to be tuned during model training, as well as additional computational cost in producing predictions. We again contrast this complex machinery with the simplicity of MOSAIKS 's approach, which

calculates label super-resolution predictions as a weighted sum of activation maps.

Conditions where label super-resolution is most easily interpretable The linear decomposition of Eq. (11) holds when using labels that represent the average or sum of values within a grid cell, such as forest cover, elevation, population density, nighttime lights, income, or road length. However, it does not hold exactly when values are transformed nonlinearly after aggregation (e.g. $\log(\sum y) \neq \sum \log(y)$).[24] In these cases, the interpretation of label super-resolution estimates requires care. Another case in which the interpretation of the sub-image predictions is difficult is when an image-level characteristic is not directly the sum of sub-image parcels. For instance, when predicting mean housing price in a grid cell, a manicured park might contribute to a higher value, yet that component of the image does not, in itself, have any associated housing price. In this case, we we would interpret the sub-image predictions as "contributions to grid cell mean housing price" (similar to the class activation maps of (*60*)) rather than the more natural interpretation as simply "a finer resolution prediction of housing price."

2.10 Global model

For our global analysis, we create a global grid, composed of roughly 420 million cells just over 1km^2 in size, using an identical structure to that described in Note 2.1 for the US. To obtain observations for our global analysis, we sub-sample 1,000,000 cells from this grid, sampling UAR from non-marine grid cells. This relatively sparse sampling of global data is due to the cost of obtaining imagery data.

One of the difficulties in sub-sampling from the global grid is that there are many grid cells where no Google imagery is available (there are negligibly few missing images in the US grid). After discarding grid cells with missing imagery from our original sample of 1,000,000 obser-

[24]This issue could be addressed – in the case of logged variables – if one obtained a geometric mean image-level outcome rather than an arithmetic mean.

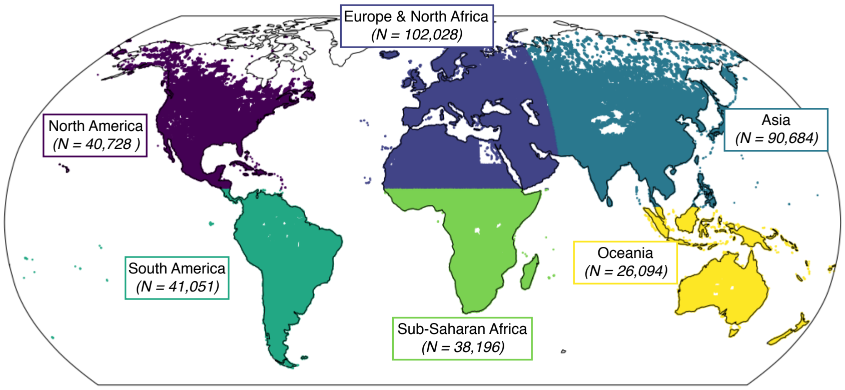

Supplementary Figure 14: **Continent samples used to solve four tasks at global scale.** MOSAIKS predictions at global scale are generated from six separate cross-validated ridge regressions using random convolutional features. Each continent model is trained on 80% of the sample size shown (N).

vations, we are left with $N = 498,063$ valid observations. After removing observations for which labeled data are missing for any of the tasks we analyze at global scale (forest cover, elevation, population density, and nighttime lights), we are left with $N = 423,476$ observations, which we use to train/validate (80%, $N = 338,781$) and test (20% $N = 84,692$) the model.

When generating features ($K = 2,048$) for our global model, we conduct featurization as described in Note 2.3. Note that to create the global features we use patches drawn randomly from the *global* sample of images, not just from within the US.

When training the global model, we follow the approach outlined in Note 2.5, solving for grid cell labels as a linear function of the random convolutional features using ridge regression and cross-validation to tune the regularization parameter λ. However, recovered regression weights are likely to differ across regions of the globe due to heterogeneity in image quality and in visual signal of task labels or their derivatives. Therefore, we divide our global sample into six continental regions before solving each task. The continents (and sample sizes used for training and testing each continent-specific model) are shown in Supplementary Figure 14.

Modeling heterogeneity using the continents shown in Supplementary Figure 14 leads to meaningful gains in performance over ignoring continent effects. As shown in the main text, the approach accounting for heterogeneity generates R^2 values of 0.85, 0.45, 0.62, and 0.49, for forest cover, elevation, population density, and nighttime lights, respectively (Figure 4). In contrast, a global model that pools all observations across the globe and solves for a single linear function of random convolutional features generates R^2 values of 0.80, 0.26, 0.48, and 0.41, for the same tasks.

2.11 Generalizing to other ACS variables

Here we demonstrate the ability of MOSAIKS to generalize rapidly across a range of new variables by replicating our primary analysis (i.e. that in Figure 2) for 12 variables from the American Community Survey, the source we use in the main text to measure income across the continental US. This survey is conducted annually across the US, tracking a diverse range of socioeconomic outcomes, from housing information to income and education. For this exercise, we select variables from the ACS that span a range of diverse outcomes and which seem likely to have at least some visible signal in daytime satellite imagery. We report performance for all tested variables.

We calculate grid cell level labels from census block group level ACS data using the the same method used for ACS income label construction outlined in Note 2.2. The resulting labels represent the area-weighted average value of the outcome across the grid cell. The ACS variables we predict are listed and described in Supplementary Table 4.

Patterns in performance across tasks could be explained by the hypothesis that some outcomes exhibit more visible features, such as age and value of housing, while other outcomes exhibit less clear visible signal, such as the percent of household income dedicated to rent.

This exercise shows the ease of generalizing MOSAIKS to new contexts. In total, training these twelve predictive models took less than 45 minutes on a workstation with ten cores (IntelXeon CPU E5-263) (Supplementary Table 8). Given the similarity in performance between MOSAIKS and other state of the art approaches documented in Note 3.1, MOSAIKS offers a relatively quick and easy way to determine how predictable a variable might be from high resolution visible satellite imagery.

3 Supplementary Note 3: Comparisons to other models

Here, we compare the predictive performance and computational cost of MOSAIKS to other approaches in the literature.

3.1 Benchmarking performance

Convolutional neural networks (CNNs) have become the default "gold standard" in many image recognition tasks (*64*), and are increasingly used in remote sensing applications (*4, 42, 5, 11, 12*) (*62, 65–68*). Simultaneously, alternative generalizable and computationally efficient pipelines have been developed that incorporate unsupervised featurization and/or a classification or regression algorithm (*44, 10, 11, 3*) (*69*). MOSAIKS is low-cost and generalizable like these latter models; however, unlike these other models, it offers accuracies for regression problems competitive with that of leading CNN architectures. Here we quantitatively assess the predictive performance of MOSAIKS relative to (a) a CNN trained end-to-end with the outcomes of interest, (b) a similarly cheap, unsupervised featurization used in place of random convolutional features in the MOSAIKS infrastructure and (c) a transfer learning approach. For (b), we use the features generated by the last hidden layer of a pre-trained variant of the CNN (trained on natural imagery). This common approach is unsupervised in that the weights of the CNN are not trained using the labels of the outcome of interest, and such an approach has been shown to have bet-

Name	Code	Description
Travel time to work	B08303	“Travel time (minutes) to work refers to the total number of minutes that it usually took the worker to get from home to work during the reference week. The elapsed time includes time spent waiting for public transportation, picking up passengers in carpools, and time spent in other activities related to getting to work.”
Percent Bachelor’s Degree	B15003	Calculated as the number of people over 25 with only bachelor’s degrees (i.e. not masters or doctorate) divided by the total number of people over 25.
Median Household Income	B19013	Median household income in the past 12 Months (2015 inflation-adjusted dollars)
Per Capita Income	B19301	Per capita income in the Past 12 Months (2015 inflation-adjusted dollars)
Percent below poverty level	C17002	Calculated as the number of people 15 years or older whose income fell below the poverty level divided by the total number of people 15 years or older.
Percent food stamp/snap	B22010	Percent household received food stamps/snap in the past 12 months.
Median income	B25071	Gross rent as a percentage of household income in the past 12 months (dollars)
Number of housing units	B25001	“A housing unit may be a house, an apartment, a mobile home, a group of rooms or a single room that is occupied (or, if vacant, intended for occupancy) as separate living quarters. Separate living quarters are those in which the occupants live separately from any other individuals in the building and which have direct access from outside the building or through a common hall. Both occupied and vacant housing units are included in the housing unit inventory. Boats, recreational vehicles (RVs), vans, tents, railroad cars, and the like are included only if they are occupied as someone’s current place of residence.”
Percent vacant	B25002	“A housing unit is vacant if no one is living in it at the time of interview.”
Structure age	B25035	Data reported is the median year structure built. We calculate structure age as 2015 – median year structure built.
Number of rooms	B25017	“For each unit, rooms include living rooms, dining rooms, kitchens, bedrooms, finished recreation rooms, enclosed porches suitable for year-round use, and lodger’s rooms. Excluded are strip or pullman kitchens, bathrooms, open porches, balconies, halls or foyers, half-rooms, utility rooms, unfinished attics or basements, or other unfinished space used for storage.”
Median house value	B25077	For owner-occupied housing units.

Supplementary Table 4: **Description of variables from the American Community Survey (ACS) used in the analysis (Figure 4).** Quoted descriptions of variables are from: https://censusreporter.org/topics/table-codes/.

ter predictive performance than many other unsupervised featurization algorithms (e.g. GIST, SIFT, Bag of Visual Words) on satellite image tasks (*69*). Previous analyses show through direct comparison that our methodology significantly outperforms ridge regression models using GIST features (*70*). Figure 15 (reproduced from (*70*)) demonstrates this comparison, describing out-of-sample performance for the prediction of housing price class for homes in Arizona.

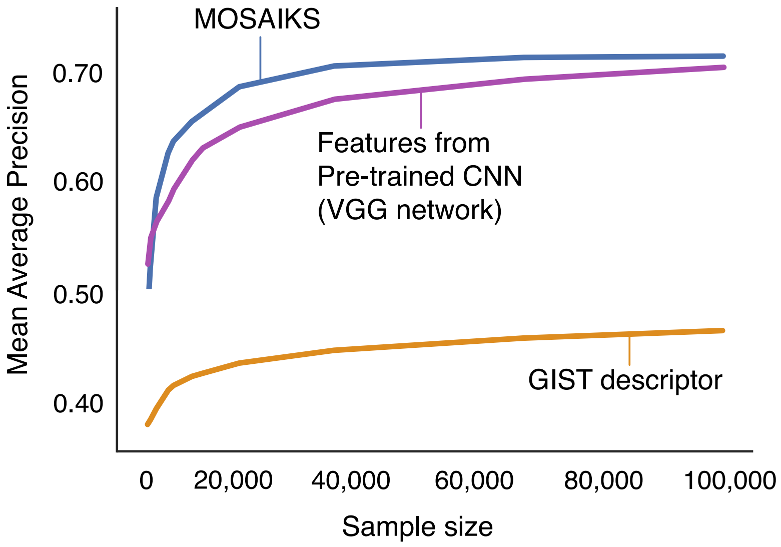

Supplementary Figure 15: **Comparison of out-of-sample performance across feature extraction techniques as a function of sample size.** Mean Average Precision is shown for the out-of-sample prediction of housing price class (low, medium, high) for all single-family home sales after 2010 in Arizona, as a function of training sample size. Three feature extraction techniques are compared: MOSAIKS (blue), a pre-trained CNN (VGG, purple), and the GIST descriptor (orange). Figure reproduced from (*70*).

These exercises compare MOSAIKS performance to that of models suited to the data availability of different prediction domains (abundant within the U.S., and relatively scarce at dispersed locations globally). A fine tuned CNN is expected to perform well in the United States, where data are relatively high quality and the sample size is large (nearly a hundred thousand observations); whereas the transfer learning approach is designed to perform well in regions where the training data are more coarse and the sample sizes are smaller (hundreds of observations). The

ability of MOSAIKS to perform on par with these approaches in each setting demonstrates its generalizability.

Comparison to a deep convolutional neural network and an alternative unsupervised featurization First, we compare the performance of MOSAIKS to that of a tuned Residual Network (ResNet) (*21*) – a common, versatile deep network architecture used in recent satellite-based learning tasks (*42*). We train this network *end to end* to predict outcomes in all seven tasks across the continental US, using as input the same imagery used by MOSAIKS.

Specifically, we train an 18-layer variant of the ResNet Architecture pre-trained on ImageNet using stochastic gradient descent to minimize the mean squared error (MSE) between the predictions and labels with an initial learning rate of 0.001 with a single decay milestone at 10 epochs, and momentum parameter of 0.9. We train the model for 50 epochs, at which point performance approaches an asymptote. The optimal values for learning rates were tuned on a validation set for the task of predicting population density. We employ a standard train/test split of 80%/20%, matching our approach when evaluating MOSAIKS.

Second, we compare MOSAIKS performance to a similarly cheap, unsupervised featurization generated by the last hidden layer of a pre-trained variant of the CNN used above, trained on natural imagery. To execute this comparison, we use the features from the last layer of a 152-layer variant of the ResNet Architecture, and then run ridge regression on these features for each task.

Supplementary Table 5 compares the holdout accuracy of MOSAIKS to both alternative approaches, demonstrating that MOSAIKS (first column) achieves performance competitive with the ResNet (second column) across all seven tasks, while providing substantially greater perfor-

Task	MOSAIKS R^2	ResNet-18 R^2	Pre-trained CNN R^2
Forest cover	0.91	0.94	0.66
Elevation	0.68	0.80	0.32
Population density	0.72	0.80	0.29
Nighttime lights	0.85	0.89	0.48
Income	0.45	0.47	0.07
Road length	0.53	0.58	0.16
Housing price	0.52	0.50	0.01

Supplementary Table 5: **Comparison of model performance between MOSAIKS, a fine-tuned ResNet-18 and a pre-trained ResNet-152.** Task-specific MOSAIKS test-set performance (first column) in contrast to: an 18-layer variant of the ResNet Architecture (ResNet-18) trained end-to-end for each task (second column); an unsupervised featurization using the last hidden layer of a 152-layer ResNet variant pre-trained on natural imagery and applied using ridge regression (third column).

mance than ridge regression run on features from the pre-trained CNN (third column). These results are shown visually in Figure 3A in the main text.

Comparison to a transfer learning approach We also compare the performance of MOSAIKS to that of a transfer learning approach in which nighttime lights observations are used to tune a CNN that was pre-trained on ImageNet. The tuned CNN is then used to extract features from the satellite images and a linear model is trained to predict the outcome of interest. This approach leverages a large number of nighttime lights observations to better learn how to extract information from satellite imagery that is meaningful to tasks that may be reflected in nighttime lights (e.g. wealth). Comparing the performance of MOSAIKS to that of transfer learning tests the value of learning these features from nighttime lights, relative to the unsupervised featurization of MOSAIKS.

We compare the performance of MOSAIKS to the transfer learning approach by replicating a subset of the analyses in (*4*) and (*13*). Using MOSAIKS, we predict wealth, electricity, mo-

bile phone ownership, education, bed net count, female body mass index, water access, and hemoglobin level in Haiti, Nepal and Rwanda; we additionally predict child weight percentile, child height percentile and child weight for height percentile in Rwanda. These variables are recorded at geo-located "cluster" locations by the Demographic and Health Survey (DHS); the survey methodology is detailed in (*4*). The variables and countries we provide performance metrics for were chosen based on the facility of obtaining and matching images and labels from the original authors and their replication code bases. We report performance for all tested variables and countries.

In this analysis, we use two MOSAIKS-based models to predict the DHS cluster labels. First, we use only the MOSAIKS random convolutional features (indicated as RCF), which we calculate for each image as detailed in Note 2.3, and then average over the 100 images associated with each DHS cluster (see (*4*) and (*13*) for details on matching images to clusters; we use the same matching approach as the original authors). In a second model, denoted MOSAIKS-NL below, we use the MOSAIKS random convolutional features along with features based on nighttime lights. The nighttime light features for each cluster are counts of the number of nightlight values that fall within a set of 19 bins, as well as the minimum, mean and maximum of the values within the image. Bins were evenly spaced on a log scale from a luminosity of 0.1 to 500 (in units of nanoWatts/cm^2/sr). We average nighttime light features for all 100 images associated with each DHS cluster, as we do for RCF.

We show results for the MOSAIKS-nighttime lights model for two reasons. First, it presents the most fair comparison to the transfer learning approach, which also leverages both nighttime lights and daytime imagery. Second, it demonstrates the ability of MOSAIKS to seamlessly combine information from different sensors – by appending their features in a linear model – to make predictions.

Training a model that uses features from multiple sensors A key benefit of the MOSAIKS approach is that it can easily combine information from multiple sensors. Recall that to train a model when using only the RCF from visual imagery we regress the outcome y_ℓ^s for each task s on features $\mathbf{x}_\ell$ as follows:

$$y_\ell^s = \mathbf{x}(\mathbf{I}_\ell)\boldsymbol{\beta}^s + \epsilon_\ell^s \tag{13}$$

And solve for $\boldsymbol{\beta}^s$ by minimizing the sum of squared errors plus an l_2 regularization term:

$$\min_{\boldsymbol{\beta}^s} \ \frac{1}{2}||y_\ell^s - \mathbf{x}(\mathbf{I}_\ell)\boldsymbol{\beta}^s||_2^2 + \frac{\lambda^s}{2}||\boldsymbol{\beta}^s||_2^2 \tag{14}$$

To include features from an additional sensor, S_ℓ, such as nighttime lights, one simply generates a new set of features, $\mathbf{z}(\mathbf{S}_\ell)$, – using the RCF algorithm or any other unsupervised featurization approach – and includes the features in the regression model, giving:

$$y_\ell^s = \mathbf{x}(\mathbf{I}_\ell)\boldsymbol{\beta}^s + \mathbf{z}(\mathbf{S}_\ell)\boldsymbol{\gamma}^s + \epsilon_\ell^s \tag{15}$$

Then, one solves for $\boldsymbol{\beta}^s$ and $\boldsymbol{\gamma}^s$ by minimizing the sum of squared errors plus individual regularization terms for each sensor:

$$\min_{\boldsymbol{\beta}^s, \boldsymbol{\gamma}^s} \ \frac{1}{2}||y_\ell^s - \mathbf{x}(\mathbf{I}_\ell)\boldsymbol{\beta}^s - \mathbf{z}(\mathbf{S}_\ell)\boldsymbol{\gamma}^s||_2^2 + \frac{\lambda_1^s}{2}||\boldsymbol{\beta}^s||_2^2 + \frac{\lambda_2^s}{2}||\boldsymbol{\gamma}^s||_2^2 \tag{16}$$

Regularizing the features from each sensor separately enables the model to treat features from individual sensors differently, which we found improves model performance. We implement a model that combines RCF and features from nighttime lights in Supplementary Figure 16. Features from additional sensors could be added to the model in a similar way.

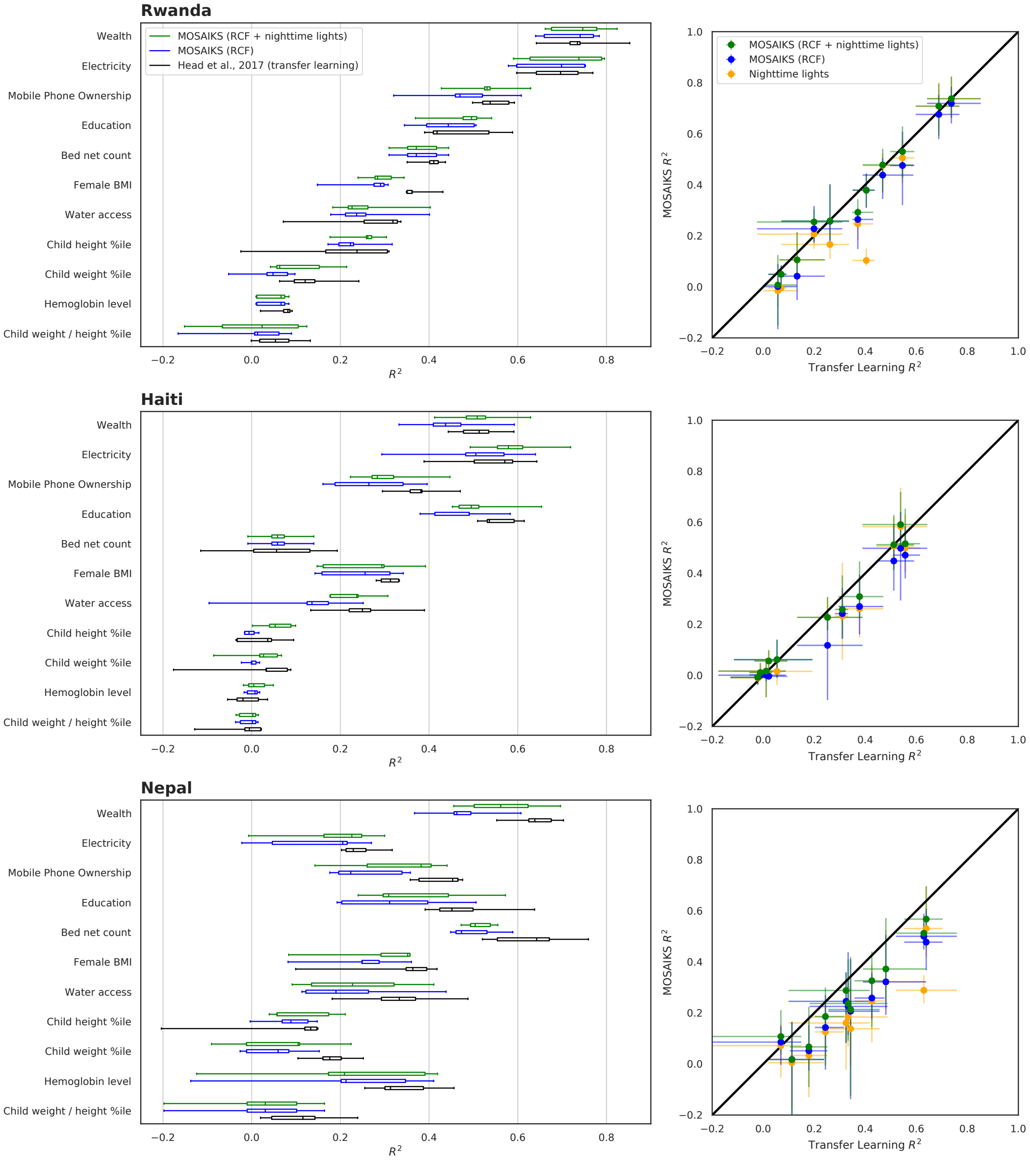

Supplementary Figure 16: **Comparison of accuracy between MOSAIKS and a transfer learning model.** Box plots (left) show task-specific performance of MOSAIKS models (random convolutional features, RCF, in blue and RCF + nighttime lights in green) in contrast to a transfer learning model (black). Box and whiskers show the performance over the 5 cross-validation folds. Scatter plots (right) show the performance of MOSAIKS models, as well as a nighttime lights-only model (orange) versus the transfer learning model performance. Each point in the scatter is the average R^2 over the 5 cross-validation folds, while whiskers indicate the full range of performance across folds.

		Method			
Country	Task	RCF	Nightlights	RCF+NL	Head et al.
Rwanda	Wealth	0.72	0.74	0.74	0.74
	Electricity	0.68	0.71	0.71	0.69
	Mobile Phone Ownership	0.48	0.51	0.53	0.55
	Education	0.44	0.48	0.48	0.47
	Bed net count	0.38	0.10	0.38	0.40
	Female BMI	0.26	0.25	0.29	0.37
	Water access	0.26	0.17	0.26	0.26
	Child height %ile	0.23	0.21	0.25	0.20
	Child weight %ile	0.04	0.11	0.11	0.13
	Hemoglobin level	0.05	0.00	0.05	0.07
	Child weight / height %ile	0.00	-0.02	0.01	0.06
Haiti	Wealth	0.45	0.51	0.51	0.51
	Electricity	0.50	0.58	0.59	0.54
	Mobile Phone Ownership	0.27	0.26	0.31	0.38
	Education	0.47	0.50	0.52	0.56
	Bed net count	0.06	0.02	0.06	0.05
	Female BMI	0.24	0.23	0.26	0.31
	Water access	0.12	0.23	0.23	0.25
	Child height %ile	0.00	0.06	0.06	0.02
	Child weight %ile	0.00	0.02	0.02	0.01
	Hemoglobin level	0.00	0.01	0.01	-0.01
	Child weight / height %ile	-0.01	-0.01	-0.01	-0.02
Nepal	Wealth	0.48	0.53	0.57	0.64
	Electricity	0.14	0.13	0.19	0.24
	Mobile Phone Ownership	0.26	0.25	0.33	0.43
	Education	0.32	0.32	0.37	0.48
	Bed net count	0.50	0.29	0.51	0.63
	Female BMI	0.25	0.16	0.29	0.32
	Water access	0.23	0.18	0.24	0.33
	Child height %ile	0.09	0.07	0.11	0.07
	Child weight %ile	0.05	0.03	0.07	0.18
	Hemoglobin level	0.21	0.14	0.21	0.34
	Child weight / height %ile	0.02	0.01	0.02	0.11

Supplementary Table 6: **Comparison of accuracy between MOSAIKS and a transfer learning model.** All columns report out-of-sample mean R^2 values, where averages are taken across five folds (ranges across all five folds are shown visually in Supplementary Figure 16). Prediction methods are the same as in Supplementary Figure 16, where "RCF" indicates random convolutional features and "Head et al." indicates the transfer learning model from ref. (*13*).

Interpretation of test accuracy comparisons Note that the performance of these models represents a reasonable lower bound on potential performance; some task-specific enhancements could be used to improve predictive power for each of these methods. For example, more layers could be added to ResNet or alternative architectures could be tested for specific tasks. In the case of MOSAIKS and the pre-trained ResNet features, more flexible regression models could be used to estimate the relationship between features and labels, such as increasing K, using a nonlinear model, or leveraging a hurdle model in tasks with a large number of zero observations. While these task-specific changes may marginally improve performance of any of these approaches, prior research on similar image recognition tasks suggests further gains for the ResNet are likely to be minimal (*71*). While the similarity of performance in Figure 3A is perhaps surprising, it is also encouraging for further research. This comparison suggests that wide, shallow networks using local-level features (analogous to random convolutional features) are as descriptive as more complex, highly optimized CNN architectures for satellite remote sensing, across many tasks.

Supplementary Figure 17 provides additional evidence that MOSAIKS and the ResNet-18 CNN display very similar patterns of predictability across tasks, as both the predictions (column 1) and errors (column 2) from the two approaches are strongly correlated. This finding suggests that MOSAIKS and the CNN may be capturing similar characteristics of the image.

To further investigate this hypothesis, we test the performance of a hybrid approach that combines MOSAIKS features with features recovered from the ResNet-18 CNN. To do so, we use the same ridge regression method from MOSAIKS; however, prior to running the regression, we concatenate the 512 features produced by the last hidden layer of the ResNet-18 to the 8,192 MOSAIKS features used throughout our analysis. In the ridge regression, we apply indepen-

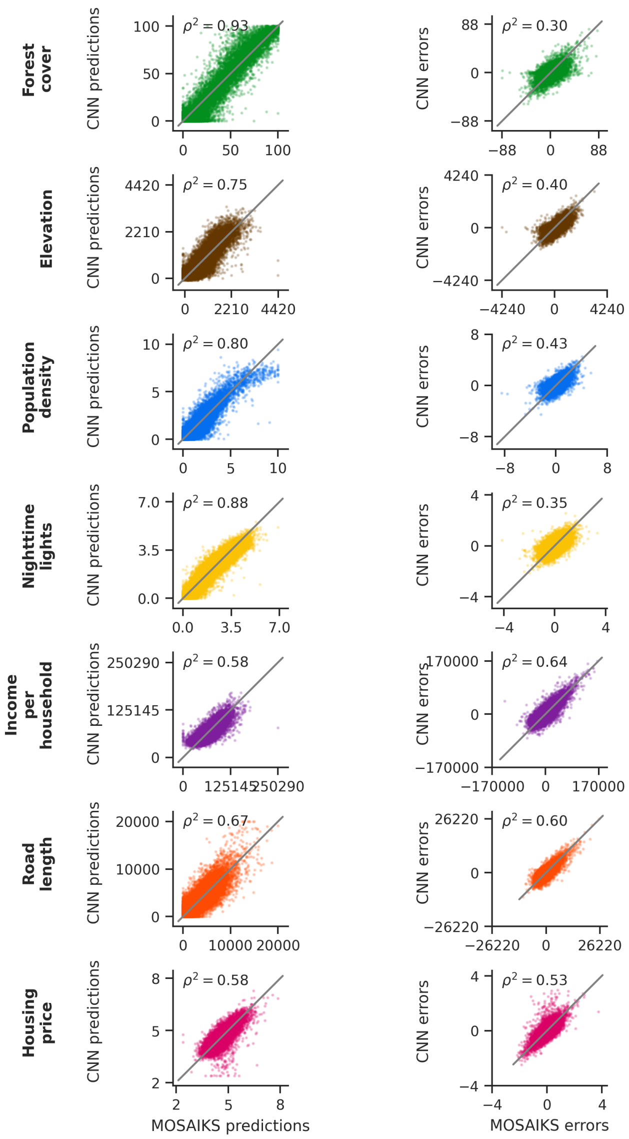

Supplementary Figure 17: **Comparison of predictions and prediction errors between MOSAIKS and the ResNet-18 CNN.** The left column shows the relationship between predictions generated by MOSAIKS (x-axis) and predictions generated by the ResNet-18 CNN (y-axis). The right column shows the relationship between prediction errors from MOSAIKS (x-axis) and prediction errors from the CNN (y-axis). In both plots, each point indicates one grid cell ($\sim$1km$\times$1km) in the holdout test set; the test set sample size is approximately 20,000 for each task, although sample sizes vary somewhat due to data availability across tasks (Note 2.5). ρ^2 values on each plot indicate the square of the Pearson correlation coefficient.

Task	MOSAIKS R^2	ResNet-18 R^2	Hybrid R^2
Forest cover	0.89	0.94	0.94
Elevation	0.68	0.80	0.81
Population density	0.71	0.81	0.81
Nighttime lights	0.85	0.89	0.90
Income	0.45	0.47	0.51
Road length	0.53	0.58	0.59
Housing price	0.53	0.49	0.58

Supplementary Table 7: **Comparison of performance of MOSAIKS, ResNet-18, and hybrid models on identical test sets**. Task-specific MOSAIKS test-set performance (first column) in contrast to: an 18-layer variant of the ResNet Architecture (ResNet- 18) trained end-to-end for each task (second column); and a hybrid model in which features produced by the last hidden layer of the trained ResNet-18 are concatenated to MOSAIKS features and included in a ridge regression (third column). Performance for all methods is evaluated on identical 10% test sets. The pre-trained ResNet-152 model is not included because it exhibits substantially lower performance (see Supplementary Table 5).

dent regularization parameters for each of the two feature sets, effectively allowing the model to rely more heavily on one or the other.

Supplementary Table 7 shows support for the hypothesis that MOSAIKS and the ResNet-18 CNN reflect similar image characteristics, as we find only a minimal performance gain from this hybrid approach (third column) for most tasks. However, we do see greater performance gains for the lowest performing tasks (income and housing price), which are also the tasks with the lowest correlation between MOSAIKS and the ResNet-18 predictions (Supplementary Figure 17). Note that in Supplementary Table 7, performance metrics for MOSAIKS and the ResNet-18 differ slightly for some tasks when compared to results in Supplementary Table 5, as the test set was defined slightly differently.[25]

[25]The hybrid approach relies on features defined by the ResNet-18 CNN, which was trained on 80% of the data. However, this method but must also use a validation set to tune the ridge regression hyperparameters. For this tuning, we extract half of the remaining 20% of the data typically used as a test set. Results are reported on the remaining, untouched, 10%. Because this test set is slightly different than that used for the individual methods in Supplementary Table 5, performance can vary slightly. Performance of all methods shown in Supplementary Table

Together, results from Figure 3A, Supplementary Figure 17, and Supplementary Table 7 are consistent with a hypothesis that both MOSAIKS and the ResNet-18 CNN are approaching the limit of information that is provided by satellite imagery for predicting the majority of outcomes we test. A human prediction baseline has not been established for these tasks, but could provide additional insight into whether there is substantial room for improvement in skill for each of these tasks. However, we suspect that in some of these tasks it will be difficult for non-expert humans to match leading SIML approaches (e.g. nighttime lights or housing prices).

3.2 Comparing costs

In practice, high computational costs can limit the use of SIML methods – especially when resources are scarce, such as in government agencies of low-income countries (*7*) or research teams and NGOs with limited budgets. Specifically designed to address this challenge, MOSAIKS scales across many research tasks by decoupling featurization from task selection, model-fitting, and prediction. The computationally costly step of featurization is done centrally on a fast computer with a graphics processing unit (GPU). Implementation of this one-time unsupervised featurization results in a roughly 6 to 1 compression of stored and transmitted imagery data with $K = 8,912$ features.[26] Individual practitioners need only download these pre-computed features, merge on labels for the task they select, and run regressions. Because features are created and stored by a central entity, the research community makes use of a cached set of computations, reducing the overall computational burden of widespread SIML and any external social costs generated by these computations (*72*). Additionally, this decoupling of task-agnostic computations from task-specific computations allows practitioners to run more diagnostic analyses on their tasks, such as those presented in Figure 3 of the main text.

7 are shown for the same 10% test set.

[26]This is calculated as: $(256 * 256 * 3)/(8192 * 4) = 6\times$ compression, where 256 * 256 * 3 integer values per image are compressed into 8192 float32 features, each of which takes $4\times$ the storage of an integer. Using 100 features gives a $500\times$ compression.

From the perspective of a user who can access pre-computed MOSAIKS features to train and validate a new task, we find that MOSAIKS is $\sim 250\times$ to $10,000\times$ faster than a state-of-the-art neural net architecture (ResNet), depending on the computational resources available to a MOSAIKS user (Supplementary Table 8). Moreover, MOSAIKS performance is competitive with the ResNet on all tasks we have studied (Figure 3A). From the perspective of the entire computational ecosystem, which bears the cost of image featurization in addition to model training and testing, we find that MOSAIKS is $5.3\times$ faster than the ResNet when solving a single task. The relative efficiency of MOSAIKS grows with the number of tasks studied because MOSAIKS features can be reused across tasks.

For the ResNet, the times in Supplementary Table 8 reflect our wall-clock time on a single Amazon EC2 instance for a single task, so that the time costs are similar to that of introducing a single new domain *ex post*. For MOSAIKS, Supplementary Table 8 includes wall-clock times on three different computational platforms, as users may have access to different resources. We show times using the same GPU as we use for the ResNet comparisons, times on a local workstation with ten cores (Intel Xeon CPU E5-263), and times on a standard laptop (MacBook Pro). For both ResNet and MOSAIKS, we report in Supplementary Table 8 model training time *after* using cross-validation to select optimal hyperparameters. For MOSAIKS, model training time on the local workstation with 10 cores is ~6.8 minutes when including cross-validation to select penalization parameters in ridge regression. The ecosystem-wide costs of featurization per task shown in Supplementary Table 8 decline as MOSAIKS becomes more widely adopted, because features can be cached centrally and distributed without modification to multiple users who are training and/or testing SIML in common locations.

We considered only one CNN architecture, which we chose because of its use in previous remote sensing applications (*4*). We did not attempt to innovate in neural net architectural design

Component	**ResNet** Time (GPU)	**MOSAIKS** Time
Training set featurization ($N = 80$k)	$\sim$ **7.9 hours**	$\sim$ 1.2 hours (GPU)
Model training		$\sim$ 2.8 seconds (GPU)<br>$\sim$ 50 seconds (10 cores)<br>$\sim$ **1.8 minutes (laptop)**
Holdout set featurization ($N = 20$k)	$\sim$ **40 seconds**	$\sim$18 minutes (GPU)
Holdout set prediction		$<$ 0.01 seconds (GPU)<br>$\sim$ 0.1 seconds (10 cores)<br>$\sim$ **0.7 seconds (laptop)**
Total cost to ecosystem	$\sim$ **7.9 hours**	$\sim$ 1.5 hours (GPU)
Total cost to user	$\sim$ **7.9 hours**	$\sim$ 2.8 seconds (GPU)<br>$\sim$ 50.1 seconds (10 cores)<br>$\sim$ **1.8 minutes (laptop)**

Supplementary Table 8: **Wall-clock times of components of MOSAIKS compared with a fine-tuned CNN.** Bold times are those that a practitioner using each method would incur (assuming MOSAIKS users have access to a standard laptop only). Model training time includes training *after* tuning for a single task for both ResNet and MOSAIKS. MOSAIKS was run using K=8,192 features. ResNet operations were run on an Amazon EC2 p3.2xlarge instance with a Tesla V100 GPU and 60GB of onboard RAM. Cost of computation on this machine is roughly $\$3/hr$. MOSAIKS operations are shown for runs on this same GPU, a local workstation with ten cores (Intel Xeon CPU E5-263), and a standard laptop (MacBook Pro).

or algorithms. While one could pursue targeted innovations in neural networks for remote sensing, such as in ref. (*66*), we emphasize that our method is currently orders of magnitude faster for the user than off-the-shelf fine-tuned CNN methods (Supplementary Table 8), does not require a GPU for prediction, and achieves competitive prediction performance (Figure 3A). There is recent work that aims to train networks to learn a "common representation" that can generalize across tasks, but this is a subject of ongoing research (*73*), requires the tasks to be known in advance, and has yet to be demonstrated or evaluated at scale.

Supplementary References

45. Alkama, R. & Cescatti, A. Biophysical climate impacts of recent changes in global forest cover. *Science* **351**, 600–604 (2016). URL http://www.ncbi.nlm.nih.gov/pubmed/26912702http://www.sciencemag.org/cgi/doi/10.1126/science.aac8083.

46. Carlson, K. M. *et al.* Effect of oil palm sustainability certification on deforestation and fire in Indonesia. *Proceedings of the National Academy of Sciences of the United States of America* **115**, 121–126 (2018). URL http://www.ncbi.nlm.nih.gov/pubmed/29229857http://www.pubmedcentral.nih.gov/articlerender.fcgi?artid=PMC5776786.

47. Elvidge, C. D., Baugh, K., Zhizhin, M., Hsu, F. C. & Ghosh, T. VIIRS night-time lights. *International Journal of Remote Sensing* **38**, 5860–5879 (2017).

48. Glenn, E. H. ACS: Download, Manipulate, and Present American Community Survey and Decennial Data from the US Census (2019).

49. Moulton, J. & Wentland, S. Monetary Policy and the Housing Market. In *Annual Meeting of the American Economic Association* (Philadelphia, PA, 2018). URL https://www.aeaweb.org/conference/2018/preliminary/paper/HTnsAQrn.

50. Gindelsky, M., Moulton, J. & Wentland, S. Valuing Housing Services in the Era of Big Data: A User Cost Approach Leveraging Zillow Microdata. In NBER (ed.) *Big Data for 21st Century Economic Statistics* (University of Chicago Press, 2019).

51. Union of Concerned Scientists. Underwater: Rising Seas, Chronic Floods, and the Implications for US Coastal Real Estate. Tech. Rep., Union of Concerned Scientists (2018). URL http://www.zillow.com/ztrax.

52. Zillow Research. zillow-research/ztrax. URL https://github.com/zillow-research/ztrax.

53. Krizhevsky, A. & Hinton, G. Learning multiple layers of features from tiny images. Tech. Rep., Citeseer (2009). URL https://www.cs.toronto.edu/~kriz/learning-features-2009-TR.pdf.

54. Coates, A., Arbor, A. & Ng, A. Y. An Analysis of Single-Layer Networks in Unsupervised Feature Learning. *International Conference on Articial Intelligence and Statistics* 215–223 (2011).

55. Recht, B., Roelofs, R., Schmidt, L. & Shankar, V. Do ImageNet Classifiers Generalize to ImageNet? In *International Conference on Machine Learning*, 5389–5400 (2019).

56. Agarwal, A., Kakade, S. M., Karampatziakis, N., Song, L. & Valiant, G. Least squares revisited: Scalable approaches for multi-class prediction. In *International Conference on Machine Learning*, 541–549 (2014).

57. Rahimi, A. & Recht, B. Random Features for Large-Scale Kernel Machines. In *Advances in Neural Information Processing Systems* (2007). URL https://people.eecs.berkeley.edu/~brecht/papers/07.rah.rec.nips.pdf.

58. Daniely, A., Frostig, R. & Singer, Y. Toward deeper understanding of neural networks: The power of initialization and a dual view on expressivity. *Advances in Neural Information Processing Systems* 2261–2269 (2016).

59. Alber, M., Kindermans, P.-J., Schütt, K. T., Müller, K.-R. & Sha, F. An empirical study on the properties of random bases for kernel methods. In *Neural Information Processing Systems* (2017). URL https://papers.nips.cc/paper/

6869-an-empirical-study-on-the-properties-of-random-bases-for-kernel-me
pdf.

60. Zhou, B., Khosla, A., Lapedriza, A., Oliva, A. & Torralba, A. Learning deep features for discriminative localization. In *Proceedings of the IEEE conference on computer vision and pattern recognition*, 2921–2929 (2016).

61. Firat, O., Can, G. & Vural, F. T. Y. Representation learning for contextual object and region detection in remote sensing. *Proceedings - International Conference on Pattern Recognition* 3708–3713 (2014).

62. Volpi, M. & Tuia, D. Dense semantic labeling of subdecimeter resolution images with convolutional neural networks. *IEEE Transactions on Geoscience and Remote Sensing* **55**, 881–893 (2017).

63. Vincent, P., Larochelle, H., Lajoie, I., Bengio, Y. & Manzagol, P.-A. Stacked denoising autoencoders: Learning useful representations in a deep network with a local denoising criterion. *Journal of machine learning research* **11**, 3371–3408 (2010).

64. Krizhevsky, A., Sutskever, I. & Hinton, G. E. Imagenet classification with deep convolutional neural networks. In *Advances in neural information processing systems*, 1097–1105 (2012).

65. Gechter, M. *et al.* The Welfare Consequences of Formalizing Developing Country Cities: Evidence from the Mumbai Mills Redevelopment. *Working paper* (2018).

66. Zhong, Y. *et al.* SatCNN: satellite image dataset classification using agile convolutional neural networks. *Remote Sensing Letters* **8**, 136–145 (2017). URL http://dx.doi.org/10.1080/2150704X.2016.1235299https://www.tandfonline.com/doi/full/10.1080/2150704X.2016.1235299.

67. Hu, W. *et al.* Mapping Missing Population in Rural India: A Deep Learning Approach with Satellite Imagery. In *Conference on Artificial Intelligence, Ethics, and Society* (2019). URL http://www.aies-conference.com/wp-content/papers/main/AIES-19_paper_157.pdf.

68. Maggiori, E., Tarabalka, Y., Charpiat, G. & Alliez, P. Convolutional Neural Networks for Large-Scale Remote-Sensing Image Classification. *IEEE Transactions on Geoscience and Remote Sensing* **55**, 645–657 (2017). URL http://ieeexplore.ieee.org/document/7592858/.

69. Cheng, G., Han, J. & Lu, X. Remote sensing image scene classification: Benchmark and state of the art. *Proceedings of the IEEE* **105**, 1865–1883 (2017). URL http://ieeexplore.ieee.org/document/7891544/.

70. Bolliger, I. *et al.* Ground Control to Major Tom: the importance of field surveys in remotely sensed data analysis. *arXiv* (2017). URL https://arxiv.org/abs/1710.09342.

71. Zoph, B. & Le, Q. V. Neural architecture search with reinforcement learning. In *International Conference on Learning Representations* (2017). URL http://arxiv.org/abs/1611.01578.

72. Strubell, E., Ganesh, A. & McCallum, A. Energy and Policy Considerations for Deep Learning in NLP. In *Proceedings of the 57th Annual Meeting of the Association for Computational Linguistics*, 3654–3650 (2019).

73. Ruder, S. An Overview of Multi-Task Learning in Deep Neural Networks. *arXiv preprint* (2017). URL http://arxiv.org/abs/1706.05098.
